# Stem Cells, Progenitor Cells, and Lineage Decisions in the Ovary

**DOI:** 10.1210/er.2014-1079

**Published:** 2014-12-26

**Authors:** Katja Hummitzsch, Richard A. Anderson, Dagmar Wilhelm, Ji Wu, Evelyn E. Telfer, Darryl L. Russell, Sarah A. Robertson, Raymond J. Rodgers

**Affiliations:** Discipline of Obstetrics and Gynaecology (K.H., D.L.R., S.A.R., R.J.R.), School of Paediatrics and Reproductive Health, Robinson Research Institute, University of Adelaide, Adelaide, South Australia, Australia 5005; Medical Research Council Centre for Reproductive Health (R.A.A.), The University of Edinburgh, The Queens Medical Research Institute, Edinburgh EH16 4TJ, United Kingdom; Department of Anatomy and Developmental Biology (D.W.), Monash University, Clayton, Victoria, Australia 3800; Bio-X Institutes (J.W.), Shanghai Jiao Tong University, Shanghai 200240, China; and Institute of Cell Biology and Centre for Integrative Physiology (E.E.T), The University of Edinburgh, Edinburgh EH8 9XE, United Kingdom

## Abstract

Exploring stem cells in the mammalian ovary has unleashed a Pandora's box of new insights and questions. Recent evidence supports the existence of stem cells of a number of the different cell types within the ovary. The evidence for a stem cell model producing mural granulosa cells and cumulus cells is strong, despite a limited number of reports. The recent identification of a precursor granulosa cell, the gonadal ridge epithelial-like cell, is exciting and novel. The identification of female germline (oogonial) stem cells is still very new and is currently limited to just a few species. Their origins and physiological roles, if any, are unknown, and their potential to produce oocytes and contribute to follicle formation in vivo lacks robust evidence. The precursor of thecal cells remains elusive, and more compelling data are needed. Similarly, claims of very small embryonic-like cells are also preliminary. Surface epithelial cells originating from gonadal ridge epithelial-like cells and from the mesonephric epithelium at the hilum of the ovary have also been proposed. Another important issue is the role of the stroma in guiding the formation of the ovary, ovigerous cords, follicles, and surface epithelium. Immune cells may also play key roles in developmental patterning, given their critical roles in corpora lutea formation and regression. Thus, while the cellular biology of the ovary is extremely important for its major endocrine and fertility roles, there is much still to be discovered. This review draws together the current evidence and perspectives on this topic.

- Introduction
- Ovarian Cell Types
- Fetal Development
  - Ovarian germ cells
  - Roles of stroma
  - Follicle formation and the origin of granulosa cells
  - Formation and the different origins of the ovarian surface epithelium
- Folliculogenesis
  - Cells of the thecal layers
  - Granulosa cells
  - Cumulus cells
- Ovulation and Corpus Luteum
  - Cell changes at ovulation
  - Cells of the corpus luteum
- Conclusions and Perspectives

## I. Introduction

The adult ovary acts primarily to support oocyte development and to secrete hormones that control puberty, the reproductive cycle, and pregnancy over the course of the finite female reproductive lifespan. These functions are associated with constant and extensive development, remodeling, and regression of the ovarian follicles and corpora lutea and involve major cellular and biochemical changes and tissue reorganization (1). Recently, many unique aspects of these processes have been discovered, and some long-held dogmas have been challenged. These processes are important because diseases of the ovary including polycystic ovary syndrome (PCOS), premature ovarian insufficiency or ovarian failure, and ovarian cancer have all been linked with alterations in these fundamental cellular processes. Additionally, attempts to promote fertility, achieve contraception, or preserve fertility by manipulating follicles are all critically dependent upon our knowledge of ovarian cellular and tissue remodeling processes. For these reasons, we review this area and focus on the origins and regulation of each cell type of the ovary during fetal development, folliculogenesis, and at ovulation and in the corpus luteum. Other aspects of follicle growth and atresia have been extensively reviewed (1–6) and are only discussed where relevant.

## II. Ovarian Cell Types

To some extent, understanding the development of the ovary can be informed by insights gained from other tissues such as the adrenal gland (reviewed in Ref. 7) and the testis (8). There is additional complexity for the ovary because, unlike most of the tissues in the body, the ovary undergoes further development starting at puberty when repeated rounds of follicle expansion, ovulation, and corpus luteum development and demise commence. In part, these hormone-driven cycles of development, remodeling, and regression reflect similar changes in other female reproductive tissues, particularly the uterine endometrium and mammary gland.

**Figure 1.**
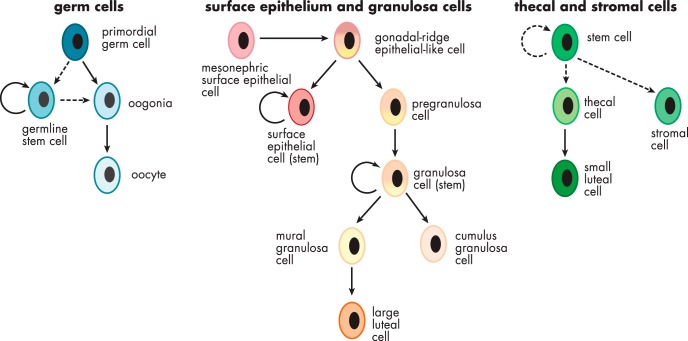
Schematic diagram illustrating the potential and known cell lineages of the ovary.

The fetal morphogenesis of the ovary is complex. Investigating this is compounded by its early origins from the mesonephros, which develops differently between males and females, and a period of bipotentiality before the indifferent gonad commits to the development into the ovary. Additionally, some ovarian cell types are derived externally, such as the primordial germ cells from the yolk sac and the immune cells, which are derived from the hematopoietic stem cells that originated from the dorsal aorta in the aorta-gonad-mesonephros region (reviewed in Ref. 9). Even the origins of some of the different somatic cell types are uncertain and may vary between species. The potential origins and lineages of ovarian cells are summarized in Figure 1, and these will be discussed in detail in the following sections.

One area of potential confusion is the terminology of progenitors and stem cells and the distinction between them. Stem cells have a number of distinct properties and express specific genes. Pluripotency is an important feature, but there are also committed stem cells that have limited potential to develop into different cell types. Tissue or adult stem cells often have restricted potential for forming different cell types. Stem cells are generally capable of dividing without the need for anchorage and are often not contact-inhibited. Non-stem cells that are not transformed need anchorage to divide, and division is inhibited when in contact with neighboring cells (10, 11). When labeled with tritiated thymidine or 5-bromodeoxyuridine, stem cells retain DNA label over many cell divisions, indicating either minimal proliferation or asymmetric use of the DNA template during replication (12, 13). Their transit-amplifying daughter cells often express telomerase (14). Because stem cells are in a unique hierarchical position, they “act as self-renewing guardians of the genome” (15) and have responses to damaging radiation that are different from other cell types (16, 17). In the ovary, both somatic and germline stem cells have been identified, and these are discussed in detail below. Although the term “germline stem cell” is used herein, this is not to indicate pluripotency such as a fertilized oocyte might exhibit, but rather the ability of the cell to undergo mitosis and consequently differentiate into oocytes.

## III. Fetal Development

### A. Ovarian germ cells

#### 1. Primordial germ cells (PGCs)

Much is known about the ontogeny of oocytes (18–20). PGCs, the carriers of genetic information for the next generation, are established very early in embryonic life. In mice, precursors of PGCs have been identified as early as embryonic day (E) 6–6.5 (for review of germ cell formation in mice, see Refs. 21–25). PGC precursors are formed under the control of signals from neighboring cells, such as bone morphogenetic protein (BMP) 2, 4, and 8, and are characterized by the expression of PR domain containing 1 (PRDM1 or BLIMP1), PRDM14, and up-regulation of Fragilis (also known as IFITM3 or interferon-induced transmembrane protein 3). At approximately E7, small clusters of PGCs, which are stabilized by E-cadherin, have been located posterior to the primitive streak in the extraembryonic mesoderm. At this stage PGCs express TNAP (nonspecific alkaline phosphatase) and DPPA3 (developmental pluripotency associated 3, also known as Stella). By E9.5, PGCs migrate to the hindgut and, directly or later via the dorsal mesentery, into the developing genital ridges. During the migration process, PGCs still express TNAP, but also OCT3/4 (octamer-binding transcription factor 3/4; also known as POU5F1), the proto-oncogene cKIT, and SSEA (stage-specific embryonic antigen) 1 and 3. At E11.5, most PGCs have arrived at the genital ridges. Human PGCs are first identified at gestational week 3 in the dorsal wall of the yolk sac in the region of the developing allantois (reviewed in Ref. 25). At gestational week 5, when the genital ridges develop, the PGCs have migrated from the hindgut to the dorsal mesentery and further laterally to colonize the two genital ridges. The colonization of the developing genital ridges by PGCs in both species is followed by sex determination (for review, see Ref. 26) and subsequent differentiation into oogonia or spermatogonia (reviewed in Ref. 25).

In addition to oogonia associating with somatic cells to form follicles, some germ cells have been identified on the surface of the ovary (27–30). These become isolated at the surface of the ovary as the penetrating stroma expands laterally below the surface of the ovary, thereby closing the once “open” ovigerous cords from the surface, leaving precursor cells of surface epithelial cells and some germ cells on the surface of the ovary (31). The fate of these germ cells is not known; however, some are lost from the surface of the ovary into the periovarian space as reported previously in humans and mice (27–30), or they could subsequently undergo cell death. It is possible that germ cells remaining at the surface become the source of the germline stem cells isolated from the surface or the outer cortex of mouse and human ovaries (32, 33), but there is no direct evidence for this.

#### 2. The debate about follicle formation later in life

For the past 60 years, a central dogma of ovarian biology has been that the entire germ cell (oocyte) pool is endowed at birth or soon after birth in some species (mouse, pig and marmoset). After this time, ovaries lose the capacity for oocyte renewal (oogenesis) (34). In 2004, studies in mice challenged the idea of a fixed ovarian reserve of oocytes and follicles, and the controversy over whether oogenesis occurs in mammals later in life was reignited (35). In the last decade, researchers identified putative germline stem cells or oogonial stem cells in postnatal ovaries of humans (33, 35), mice (32, 36), and rats (37), and many controversies arose and have polarized debates, which will be discussed further here. We separate the issues into two major topics. First, we discuss the debate about oogenesis and follicle formation in adult life. Second, we discuss the evidence that ovarian cells exist with mitotic potential and ability to be differentiated into oocytes in vitro. These two issues are separate in our minds, but clearly the first issue has clouded rational debate about the second.

Johnson et al (35) were the first to suggest “the renewal of germ cells in postnatal mice ovaries” after examining changes in follicle numbers from birth to adulthood. In a subsequent publication, Johnson et al (38) showed the expression of germline markers in bone marrow-derived cells. Furthermore, bone marrow and peripheral blood transplantations restored the oocyte production in wild-type mice sterilized by chemotherapy and ataxia telangiectasia-mutated mice. The authors concluded that bone marrow and peripheral blood might be a potential source of female germ cells that could sustain oocyte production in adulthood. However, this suggestion (38) was not supported by a parabiosis experiment (39) in which the vasculature of wild-type mice was surgically connected to that of transgenic mice expressing green fluorescent protein (GFP) under the control of the β-actin promoter. Despite observing high levels of blood cell chimerism, no GFP-positive germ cells were ovulated in the nontransgenic mice. GFP-positive cells detected in the cumulus mass associated with ovulated oocytes in the wild-type mice were identified as hematopoietic cells by staining for CD45.

Subsequently, another study examined the effects of bone marrow transplantation from TgOG2 transgenic mice with germline-specific expression of GFP (*Oct4*-GFP) into recipient mice depleted of their follicles by busulfan and cyclophosphamide treatment (40). Bone marrow-derived germ cells were observed in primordial and immature growing follicles, which did not mature to the ovulatory stage. Furthermore, it was shown that the bone marrow-derived germ cells were not CD45-positive monocytes as suggested by Eggan et al (39), and *Oct4*-GFP is not entirely germ cell-specific, with expression also detected in other adult stem cell populations and tumors (41). In addition, there is still the possibility that GFP-positive cells observed in the recipient mice by Lee et al (40) were macrophages because they did not have the typical morphology of oocytes, and *Oct4*-positive macrophages have been observed before in atherosclerotic plaques in the rabbit (42).

These discordant observations and conclusions may be reconciled by suggestions that transplanted bone marrow-derived or blood-borne leukocytes do not replace germ cells, but instead nurture and support their development and recovery from irradiation or chemotherapy and/or protect against autoimmunity (41). A number of observations implicate immune cells in germ cell support and follicle development. A key population of T cells in rodent (43) and human (44, 45) ovaries protects the oocyte from autoimmune destruction. The protection-conferring population is CD4^+^CD25^+^FOXP3^+^ Treg cells. Cells from females are intrinsically more potent suppressors than cells from males (46). The gender-specific effect can be reversed if males are grafted with ovaries before recovery and transfer of Treg cells (47). This shows the antigen-specific nature of the Treg suppressive activity and the necessity for the persistent presence of the cognate tissue antigen in generating the ovary antigen-specific Treg cells. Operational failure of the normal immune regulatory mechanisms in the ovary and its draining lymph nodes, particularly loss of immune suppressive regulatory T cells (Treg cells), may be instrumental in causing premature ovarian insufficiency in some women (48), demonstrating the key role of Treg cells in sustaining normal follicle function.

Further evidence opposing a hematopoietic stem cell source of oocytes came from a study using “a molecular clock” approach to estimate the number of mitotic divisions a cell had undergone since arising from the zygote (49). This approach used the genetic information encoded in somatic mutations to reconstruct cell lineage trees. It is based on the idea that the spontaneous mutations in DNA can be used as a molecular clock, effectively counting the number of mitotic divisions a cell has undergone since the zygote (denoted as “depth”). The pattern of somatic mutations in multiple loci can reveal the lineage relations among individual cells. Using this approach, the authors found evidence to support the “production-line hypothesis” of oocyte activation where the first oocytes to be ovulated during life are those that entered meiosis first (50). Importantly, they found that the “depth” of oocytes was different from both mesenchymal and hematopoietic bone marrow stem cells (49). Hence, they found no evidence that oocytes were derived from bone marrow cells.

Thus, by way of summary and based on the current evidence, most researchers of this area are not convinced by any of the current data or claims that oogenesis or follicle formation occurs later in life. They believe that oocytes develop from PGCs prenatally or early postnatally in some species. Thus, the original dogma that the entire germ cell (oocyte) pool is endowed at birth or soon after birth in some species still holds true.

#### 3. Isolation of ovarian cells demonstrating germline potential after in vitro manipulation

A major turning point in this new field came in 2009 when a population of cells that were mitotically active in vitro and could be manipulated to demonstrate germline characteristics was isolated from both immature and adult mouse ovaries (32). The isolation of these cells, however, did not demonstrate in any way that they are involved in oogenesis or follicle formation in later life. We treat the discussion of the biology of these cells separately from issues surrounding whether or not they can develop into oocytes in vivo.

The ovarian-sourced cells were distinct from bone marrow-derived cells, and these cells showed stable expression of germline markers (*Oct4*, *Mvh*, *Dazl*, *Blimp1*, *Fragilis*, *Stella*, and *Rex1*) (32). It was shown that these cells could be putative ovarian germline stem cells using transplantation models to repopulate the oocyte pool in chemotherapy-damaged mouse ovaries. New oocytes were formed and were capable of fertilization leading to the birth of live offspring carrying a traceable genetic marker (GFP) introduced into the cells before transplantation. Mating of this F1 generation with wild-type mice produced transgenic F2 offspring, which inherited the GFP transgene transmitted through the germline (32).

It has been suggested that the ovarian surface epithelial layer might be a source of germline stem cells because immunohistochemistry revealed cells that were double-positive for both mouse vasa homolog (MVH) and 5- bromodeoxyuridine, a proliferation marker (32, 35). Pacchiarotti et al (51) used a female transgenic mouse model that expressed GFP under the control of the *Oct4* promoter, and they located GFP-positive cells on the ovarian surface epithelium in postnatal mice. Isolated GFP-positive cells were stable in culture for up to 1 year, expressing germ cell-specific markers (GCNA [germ cell nuclear antigen], cKIT, MVH) and maintaining telomerase activity. The culture of these germline stem cells with granulosa cells of neonatal mice in hanging drops resulted in the formation of follicle-like structures, but their functionality has not been investigated further. Subsequently, Zhang et al (36) transfected short-term cultured germline stem cells from neonatal and adult mice expressing GFP and transferred these into chemotherapy-pretreated recipient mice, which produced transgenic F1 and F2 offspring. Furthermore, transfection of cultured germline stem cells with recombinant viruses carrying Oocyte-G1, a protein with potential involvement in ovarian follicular development (52), or *Dnaic2* (mouse dynein axonemal intermediate chain 2), or liposome-mediated transfection with an Oocyte-G1 knockdown vector resulted in the production of heterozygous offspring after transplantation into chemotherapy-pretreated mice, allowing the study of the role of these proteins. As a control in these experiments (36), no transgenic offspring were observed after transplantation of short-term cultured and GFP-transfected oocytes, providing proof that the transgenic offspring observed after transplantation of GFP-positive germline stem cells were not from oocytes.

Comparisons of gene expression profiles between embryonic stem cells, PGCs, freshly isolated germline stem cells, and cultured germline stem cells from adult mice showed that the profile of PGCs had great similarity to embryonic stem cells, whereas fresh germline stem cells lacked the expression of the pluripotency-associated genes *Zfp296* (encoding zinc finger protein 296), *Nr0b1* (nuclear receptor subfamily 0 group B member 1), *Utf1* (undifferentiated embryonic cell transcript factor-1), *Nanog*, and *Sox2* (SRY box 2) (53). Cultured germline stem cells (23rd passage) resembled PGCs as *Zfp296*, *Utf1*, *Nanog*, and *Sox2* were expressed. Interestingly, these cultured germline stem cells also weakly expressed *Stra8* (stimulated by retinoic acid 8), a marker of meiotic entry. Park et al (54) observed that less than 1% of approximately 2.5 × 10^4^ seeded germline stem cells spontaneously differentiated into oocyte-like cells that expressed the meiotic marker *Stra8*. The addition of BMP4, which plays a role in the generation of PGCs in mouse embryos (55), to the germline stem cell cultures increased the total number of oocytes 2-fold and significantly increased the expression of *Stra8* and *Msx1* (muscle segment homeobox 1) and *Msx2* (54), which are BMP-responsive genes in human and mouse fetal ovaries (56, 57).

Criticisms of the study by Zou et al emerged (32), questioning the isolation protocol and the purity of the cells (58). To address these concerns, in 2012 White et al (33) described an improved fluorescence-activated cell sorting (FACS)-based protocol for the isolation and purification of germline stem cells from adult mouse ovaries and confirmed prior work that the primitive germ cells obtained can generate fertilizable oocytes and embryos. In the same study, germline stem cells were purified from adult human ovaries, propagated in vitro, and shown after injection into human ovarian cortical pieces to generate what appeared by morphology and genetic markers to be immature oocytes that had become enclosed by granulosa cells to form follicles.

The approach of using DDX4/MVH for the isolation and purification of germline stem cells has been criticized because DDX4, a RNA helicase, is usually expressed in the cytoplasm of germ cells (59). However, the use of two DDX4 antibodies, one against the C terminus and the other against the N terminus, by White et al (33) resulted in the isolation of DDX4-positive cells by FACS only with the antibody against the C terminus, whereas a previous cell permeabilization step led to DDX4-positive cells with both antibodies. Cells isolated without permeabilization expressed additional germline markers such as *Prdm1*, *Dppa3*, *Dazl*, *Tert* (telomerase reverse transcriptase), and *Ifitm3* (Fragilis), but not oocyte-specific markers such as *Zp3* (zona pellucida sperm binding protein 3), *Nobox* (newborn ovary homeobox protein), or *Gdf9* (growth differentiation factor 9). This suggests the existence of “immature” germline cells in the ovary that express DDX4 or domains of DDX4 on the cell surface. It has been proposed that DDX4 is silenced in undifferentiated germline stem cells by insertion into the cell membrane, and after commitment to the oocyte fate, DDX4 is no longer externally expressed (53). An isolation method for mouse germline stem cells using antibodies to Fragilis, which is a known transmembrane protein (60), for antibody-assisted magnetic-bead sorting has been established (61) and may offer greater efficiency of isolation.

The existence of female germline stem cells as contributors to follicle formation remains to be further studied. Using multiple fluorescent Rosa26^rbw/+^;Ddx4-Cre germline reporter mice, Zhang et al (62) reported that DDX4-positive cells isolated from adult ovaries were not mitotically active, whereas they were when isolated from testes. It has to be noted that there was no distinction between germline progenitors and oocytes using this genetic approach, and it appears that most experiments concentrated on cells of the size of meiotic oocytes. These results could indicate that the isolated cells were oocytes. A key difference is that Zhang et al (62) did not follow the protocol from Zou et al (32) and White et al (33). These and related issues have been raised again (63) with claims that there are mitotically active germline stem cells in the ovary. Lei and Spradling (64) reported that the neonatal pool of primordial follicles was stable enough to sustain adult oogenesis without renewal of the pool. However, they did not trace individual germline cell development.

Recently it has been shown that female germline stem cells isolated from neonatal and prepubertal mice can be converted into pluripotent embryonic stem-like cells when cultured under certain conditions (65). Furthermore, female germline stem cells show morphological and molecular characteristics, as shown by gene expression profile, similar to male germline stem cells/spermatogonial stem cells (66).

In summary, germline stem cells appear to exist in ovaries, and they can be isolated and manipulated in vitro and give rise to offspring upon transplantation. They have been isolated independently by at least two research groups and from a number of species (human, mouse, rat). However, the physiological relevance of these cells to adult ovarian function and fertility, if any, remains to be determined. There are claims and counterclaims that these cells are mitotically active in the ovary, but there is no evidence that they contribute to oogenesis or follicle formation in vivo. Their location is either on or near the surface of the ovary, and it has been suggested previously that they could have been derived from oogonia trapped on the surface of the ovary during development (31). Thus, although at present there remains controversy over the biological significance of these cells, their identification and isolation clearly represents a significant advance with the future potential to change infertility treatments, and possibly even to alleviate nonreproductive consequences of the loss of ovarian function, as well as being a valuable model for understanding germ cell development.

#### 4. Other reports of germline stem cells

There are other reports on germline stem cells; these reports appear to be targeting cells different from those discussed in *Section III.A.3*, and we summarize them briefly here. Recent publications have reported that 2- to 4-μm small round cells, isolated from ovarian surface epithelium scrapings of postmenopausal women and women with primary premature ovarian insufficiency, spontaneously differentiate into oocyte-like cells in the presence of follicular fluid or estrogenic stimuli (67–71). The oocyte-like cells expressed pluripotency (*OCT4*, *SOX2*, *NANOG*, *NANOS*) and germ cell markers (*cKIT*, *VASA*, *STELLA*, *SCP1–3* [synaptonemal complex protein 1–3]). The authors compared the small cells originally isolated from the surface epithelium to very small embryonic like (VSEL) stem cells (70). VSEL cells have been proposed to be present in the ovarian surface epithelium of species such as human, mouse, sheep, rabbit, monkey, and marmoset (72–75), based on detection of germ cell markers (cKIT) and pluripotency markers (OCT4, NANOG, SOX2, SSEA4) by immunohistochemistry and RT-PCR in ovarian tissue biopsies or cultures of scraped ovarian surface. It was hypothesized that germline stem cells are descendants of VSEL cells and would differentiate into the observed oocyte-like cells in culture. However, these studies have not used any antibody-related isolation methods to obtain pure VSEL cells or tracing methods to show that VSEL cells from the ovary are germline stem cells. Many of the attributes of the proposed ovarian VSEL cells remain unclear.

In contrast to other theories on the origins of germline stem cells, Bukovsky and colleagues hypothesized that putative germ cells can originate by differentiation from ovarian surface epithelial cells in adult rodent (76), monkey (77), and human (77–79) ovaries. The proposed mechanisms by which epithelial cells do this and how they associate with newly developing granulosa cells involved a proposed complex set of cell relocations within the ovary and alterations in cell phenotypes (reviewed in Ref. 80). Part of these theories is based on immunohistochemical staining for the meiotic marker synaptonemal complex protein 3 (SYCP3), zona pellucida proteins, or PS1, a carbohydrate antigen of the zona pellucida, in ovarian tissue sections or cultures derived from scraped ovarian surface cells. Neither the ovarian tissue sections nor the isolated surface cells with an oocyte phenotype have been characterized for germ cell markers such as OCT4, MVH, DAZL, or SSEA4, nor is there proof of functionality of these cells as proposed.

In summary, these reports of germline stem cells in the ovary discussed in this section lack the proof that the cells being examined have any germline stem cell characteristics.

### B. Roles of stroma

**Figure 2.**
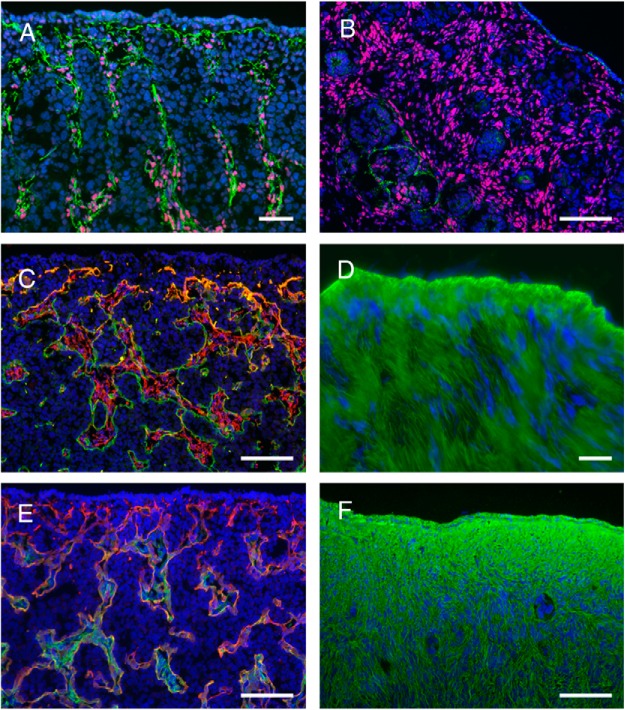
Localization of extracellular matrix components in the stroma of fetal and adult bovine ovaries. A and B, Localization of fibrillin 3 (green) with the stromal cell marker COUP-TFII/NR2F2 (red) in fetal ovaries at gestational days 96 (A) and 182 (B). C, Fibrillin 1 expression (red) and components of laminin 111 (green) in fetal ovary at gestational day 79. D, Fibrillin 1 (green) localization in adult ovary. E, Localization of decorin fibers (green) and components of laminin 111 (red) in fetal ovary at gestational day 79. F, Localization of decorin fibers (green) in adult ovary. Nuclei are counterstained with DAPI (4',6-diamidino-2-phenylindole; blue). Scale bars, A and D, 25 μm; B, C, E, and F, 50 μm. [Panels A and B were reproduced from K. Hummitzsch et al: A new model of development of the mammalian ovary and follicles. *PloS One*. 2013;8:e55578 (31), with permission, and staining in panels C, E, and F was conducted as reported previously in the same article. Panel D was reproduced from M. J. Prodoehl et al: Fibrillins and latent TGFβ binding proteins in bovine ovaries of offspring following high or low protein diets during pregnancy of dams. *Mol Cell Endocrinol*. 2009;307:133–141 (98), with permission. Elsevier.]

It is becoming apparent that the stroma plays a number of pivotal roles in the ovary. The extent of stroma and its marker (COUP-TFII/NR2F2) and extracellular matrices (fibrillins and decorin) is illustrated in Figure 2. During the formation of the ovary, the stroma penetrates from the mesonephros into the gonadal ridge/ovarian primordium, then composed of gonadal ridge epithelial-like (GREL) cells (58, 81) and PGCs. During penetration, the stroma branches, and this process creates areas of stroma alternating with areas of GREL cells/germ cells and hence produces the ovigerous cords, which are composed of GREL cells and germ cells. These cords are therefore initially “open” to the surface. The penetrating stroma has been observed previously and described as “cell streams” (82). At all times, there is continuous basal lamina between the stroma and the ovigerous cords, between the stroma and follicles, and between the stroma and the surface epithelium. The composition of the basal laminas in these locations is identical. They contain components of laminin 111, collagens type IV and XVIII, perlecan, and nidogens 1 and 2. This supports the notion that cords, follicles, and surface epithelium are formed or compartmentalized by the penetrating stroma, and this highlights an underappreciated role of the stroma in the ovary.

Additionally, the stroma is important because it penetrates what will become the cortex of the ovary. The penetrating stroma contains a vascular capillary bed, and it thereby provides a blood supply to the cortex. Hummitzsch et al (31) reported that “when the stroma penetrates into the gonadal ridge/ovarian primordium, it contains endothelial cells assembled into mature capillaries surrounded by a subendothelial basal lamina. Thus this capillary network of the ovarian cortex is not likely formed by the vascularization process but rather by sprouting or splitting forms of angiogenesis (83) allowing expansion of the existing capillary network derived directly from vasculature in the mesonephros.” Thus, once penetration of stroma into the ovary primordium has commenced, the growth and expansion of a capillary network within the stroma would occur within the ovary by angiogenesis. This appears to happen in mouse where “few endothelial cells crossed the border between the mesonephros and the XX gonad” (84) and “the XX gonad recruits vasculature by a typical angiogenic process” (85). It has been suggested that vasculogenesis also occurs (84, 86); however, this is likely to be an early event, and to what degree it contributes to overall vascular development is unclear. Vascularization of the presumptive ovary happens significantly later than in the presumptive testis and is less pronounced, making the vascular structure one of the first distinguishing morphological features of the two genders (85). Lymphatics do not enter the developing ovary until much later. In the mouse, lymphatic vessels in the ovary are absent until around postnatal day 10, the time when the first wave of growing follicles becomes estrogenic (87). As the follicles continue to grow, highly branched lymphatic vessels are recruited to the theca and stromal layers around each follicle, and as a result of this process, the ovarian lymphatic network is established (88, 89). It is subsequently remodeled to accommodate the growth of each new follicle wave throughout the reproductive lifespan (88, 89). Blockade of VEGF-R3 (vascular endothelial growth factor [VEGF] receptor 3) signaling prevents neolymphangiogenesis around developing follicles, reducing follicle viability and hormone secretion and leading to poor embryonic developmental competence (90).

Aberrant stromal activity may also be important in human conditions such as PCOS. It is well known that PCOS ovaries have increased numbers of antral follicles, but it is less well appreciated that they also have substantially more tunica albuginea containing more collagen, and they also have increased thicknesses of cortical and subcortical stroma (91). This fact has been known since the early reports of PCOS (92, 93); however, these features of PCOS ovaries had not received much attention until recently when it was discovered that the fibrillin 3 gene, located in a genomic region associated with PCOS (94), is expressed in the penetrating stroma in human and bovine ovaries in the first trimester (95). Fibrillins regulate TGFβ activity in tissues (96, 97), and in turn, TGFβ stimulates stromal fibroblast replication and collagen deposition, which are increased in the PCOS ovary. Thus, the regulation and role(s) of ovarian stoma clearly warrant further investigation.

The tunica albuginea is not as thick in the ovary as in the testis. It is variable in thickness from one location to another in the ovary and appears to have some degree of zonation (98). It is not vascularized (99) and contains much structural collagen and other extracellular components and in differing amounts to the cortical stroma below it (31, 95, 98). The tunica albuginea is derived from the stroma that penetrated the ovary in the cell streams to just below the surface of the ovary as described previously (31). What initiated the changes in the stroma to form the tunica albuginea is not known. Additionally, the tunica albuginea also undergoes a cycle of cell death and tissue repair at the apex of ovulating follicles (100).

### C. Follicle formation and the origin of granulosa cells

**Figure 3.**
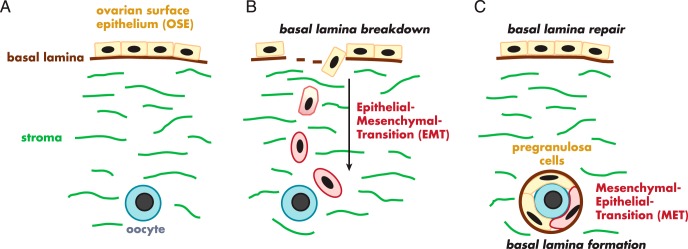
Illustration of the conceptual processes needed to derive granulosa cells from the ovarian surface epithelium. There has been no discussion of these processes in the literature or any evidence to identify that they occur. Surface epithelial cells with a basal lamina and stromal interface (A) would first need to undergo an epithelial-mesenchymal transition (B) to break through the surface epithelial basal lamina and to become migratory and migrate to the oogonium (B). They would then need to undergo a mesenchymal-epithelial transition to form epithelial granulosa cells of follicles all enclosed by the follicular basal lamina (C).

The origins of somatic granulosa cells attract considerable conjecture. Granulosa cells were originally considered to be derived from the mesonephric tubules and more recently from the ovarian surface epithelium (reviewed in Refs. 19 and 20). The mesonephros is a complex structure with many different cell types, including stromal cells, endothelial cells, and the different epithelia associated with its nephrons. In mammals, the mesonephros is a transient organ during fetal development, and it develops differently between males and females (101–107) (for reviews see Refs. 108 and 109). In females, it contributes tubules to the hilum and medulla of the ovary, and these persist into adulthood, referred to as the rete ovarii. The evidence that these structures give rise to granulosa cells came from early observations that rete ovarii can have a close association with oocytes (110, 111). This was further strengthened by demonstration that the presence of rete ovarii correlated with the onset of meiosis (112) and follicle formation (113). Subsequently it was suggested that cells derived from the ovarian surface epithelium give rise to the granulosa cells during follicle formation (82, 114). Part of the confusion about the origins of granulosa cells from surface epithelial cells could be clarified by the use of correct terminology. A simple classic epithelium, such as the mature ovarian surface epithelium, consists of a single layer of epithelial cells with an underlying basal lamina at the interface with stroma. If granulosa cells are derived from classic ovarian surface epithelial cells, as opposed to cells located at the surface (no underlying basal lamina and no epithelial-stroma interface), then presumably the surface epithelial cells would need to undergo an epithelial-mesenchymal transition followed by a mesenchymal-epithelial transition, as illustrated in Figure 3—a process for which no evidence exists. With the model of GREL cells as proposed in the bovine (31), the gonadal ridge/ovarian primordium is initially not covered by a classic surface epithelium; instead it is covered by GREL cells that are located at the surface. Thus, we suggest that a way forward is to interpret existing publications to mean that granulosa cells are derived from cells on the surface of the ovary rather than specifically from a classic surface epithelium.

**Figure 4.**
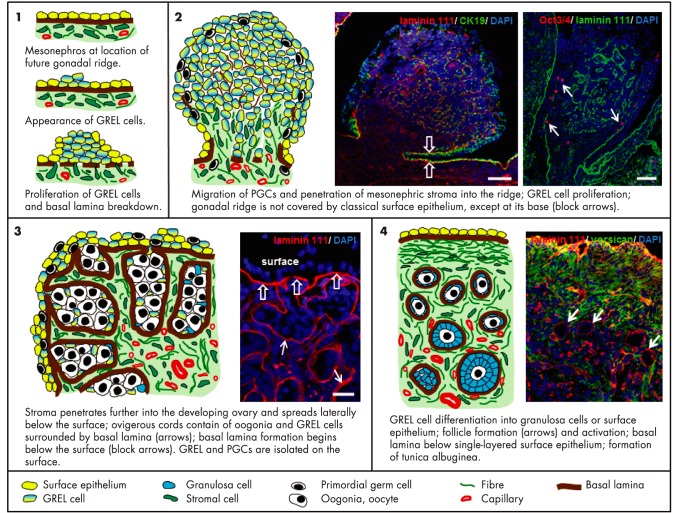
Schematic diagram of ovarian development. [Reproduced from K. Hummitzsch et al: A new model of development of the mammalian ovary and follicles. *PloS One*. 2013;8:e55578 (31), with permission.]. Abbreviations: CK19, cytokeratin 19; DAPI, 4',6-diamidino-2-phenylindole.

A more recent examination of bovine ovarian development suggests that granulosa cells are not derived from differentiated ovarian surface epithelial cells. Instead, both the apical ovarian surface epithelium and the granulosa cells arise from a precursor population of GREL cells (31) (Figure 4). GREL cells are postulated to be derived from cells of the surface of the mesonephros (31), which replicate to form the genital ridge/ovarian primordium into which the PGCs migrate. Soon afterward, cords of stromal cells referred to as “cell streams” penetrate the primordium from the underlying mesonephros, partitioning the developing ovary into irregularly shaped ovigerous cords composed of GREL cells and PGCs/oogonia. A basal lamina is formed and separates the stromal cells from the ovigerous cords, which at this stage contain GREL cells and oogonial “nests” or small syncytial groups of germ cells that have not completed cytokinesis (31). As development progresses, apoptosis of oogonia occurs, and the oogonial nests are reduced to individual oocytes surrounded by a finite number of GREL cells to form primordial follicles. The basal lamina, which had previously separated the ovigerous cords from the surrounding stroma, now surrounds individual follicles. This interaction with the stroma is a key aspect of follicle formation that has received little attention. There is thus a complex three-way interaction between the oogonia, GREL cells, and the stroma that ultimately determines the number and potentially the quality of follicles with which the ovary is endowed.

The formation of follicles commences from the medullary end of the cords and progresses to the surface as the stroma penetrates toward the surface. This progression of maturation has been observed in fetal human (115–117), cattle (31, 118, 119), sheep (114, 120, 121), mouse (122) and postnatal rat ovaries (123, 124). It is accompanied by changes in maturation markers in the oocytes (from OCT3/4, to deleted in azoospermia-like [DAZL], and then to VASA [also known as mouse vasa homolog, MVH], or DEAD [Asp-Glu-Ala-Asp] box polypeptide 4 [DDX4]) and in the GREL/granulosa cells (from replication markers to expression of FOXL2 [forkhead box L2]). The behavior of the stroma appears to be pivotal for formation of follicles, and it is noteworthy that the follicles form first in areas where the stroma first contacts and partitions the ovigerous cords. This gradient of development may have consequences for follicle activation later in life where it has been recognized for many years that the first follicles to initiate growth are those that were formed the earliest, sometimes called the “first in, first out” or the “production line” hypothesis (124, 125). This concept has been extended in the mouse for the existence of two waves of primordial follicle activation constrained to their medullary or cortical locations (126–128). The first wave of follicle activation occurs in the medulla, and these follicles contribute to most of the growing follicle pool until approximately postnatal day 45. Thereafter there is a decline such that they constitute only 2.4% of growing follicles by postnatal day 90 (127). The cortical primordial follicles are activated later and provide fertility throughout adulthood (126). The two waves of follicle activation in the mouse were discovered because differential expression of cell markers in precursor granulosa cells was observed and molecular methods for inducible marking of cells were available. These latter methods are currently not available in other species, and thus it not easy to confirm whether there are similar waves in other species. It would be useful now to determine how staccato-like these waves are or how much of a continuum they represent. This could be achieved by increasing the number of periods of inducible marking of granulosa cells during gestation and early postnatally. Additionally, as suggested (126), it is important to determine whether differences in these waves of follicles are due to “their different origins or to environmental factors, such as gonadotropin levels and diet, which differ in prepubertal and adult animals.”

The medullary follicles contain granulosa cells arising from precursor cells that expressed FOXL2 in the fetal ovary (126–128). Recently, it was shown that granulosa cells from cortical follicles are derived from LGR5 (leucine-rich repeat-containing G protein-coupled receptor 5; also known as GPR49 or GPR67)-positive cells, which are located in the cortex and at the ovarian surface (128). It was suggested that the FOXL2- and LGR5-expressing pregranulosa cells are two distinct populations because coexpression of these two markers between E13.5 and E18.5 was not observed (128). Eventually, all granulosa cells express FOXL2 (129). Mork et al (126) concluded that “while the granulosa cells of the medulla and cortex can be classified into two separate populations, they are likely the descendants of a single progenitor source, born at different stages of development,” which would be compatible with the GREL cell model in some aspects.

### D. Formation and the different origins of the ovarian surface epithelium

**Figure 5.**
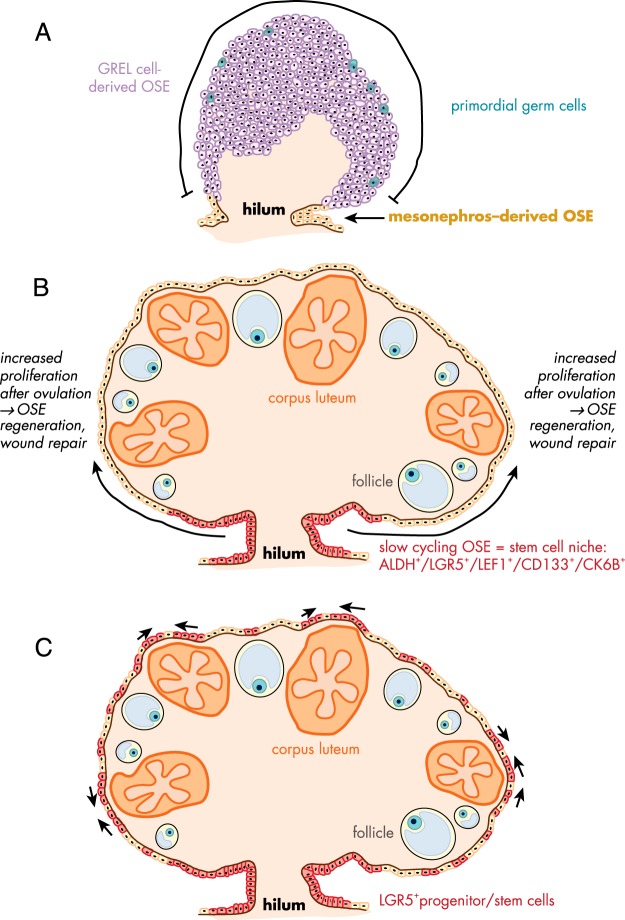
Schematic diagram of the proposed development and repair of ovarian surface epithelium. A, In early fetal ovarian development, the ovarian surface epithelium (OSE) in the hilum region is derived from the mesonephros, whereas the remaining surface of ovary is covered by GREL cells, which later differentiate to surface epithelial cells or granulosa cells. [Adapted from K. Hummitzsch et al: A new model of development of the mammalian ovary and follicles. *PloS One*. 2013;8:e55578 (31).] B, Flesken-Nikitin et al identified an ALDH-, LGR5-, LEF-1-, CD133-, and CK6B-expressing stem cell niche in the hilum region of adult mice ovaries, which is responsible for the OSE repair after ovulation and is susceptible to malignant transformation. [Adapted from A. Flesken-Nikitin et al: Ovarian surface epithelium at the junction area contains a cancer-prone stem cell niche. *Nature*. 2013;495:241–245 (136).] C, A recent study in adult mice identified LGR5-positive OSE stem cells not only in the hilum region but also along the remaining ovarian surface as small clusters, mainly near ovulating follicles and on the apical side of corpora lutea. [Adapted from A. Ng et al: Lgr5 marks stem/progenitor cells in ovary and tubal epithelia. *Nat Cell Biol*. 2014;16:745–757 (137).] Abbreviations: ALDH, aldehyde dehydrogenase; LEF1, lymphoid enhancer-binding factor 1; CD133, cluster of differentiation 133; CK6B, cytokeratin 6B.

The mature ovary is covered by a single layer of flat to cuboidal epithelial cells, the surface epithelium. This layer constantly undergoes morphological changes, particularly during the repair of the ovarian surface after the rupture of the follicle wall during ovulation (130). The physiology of ovarian surface epithelium; its regulation by hormones, growth factors, and cytokines; and its involvement in the ovulatory process have been reviewed previously (131–133), and Figure 5 illustrates more recent theories about the origins of these cells.

Previous literature implied that the surface epithelium originates from the mesoderm-derived epithelial layer, which lines the intraembryonic coelom and the area where the gonad is formed. The gonadal blastema is partly formed by proliferation of the surface epithelial lining (134) (reviewed in Ref. 131). However, we observed recently that when the bovine fetal ovary is first formed, it is not covered by a defined surface epithelium underlaid by a basal lamina at an interface with stroma, as observed in adult ovaries, except at the base of the ovary where it arises from the mesonephros (31, 81). We hypothesized that the early developing ovary is composed of a cluster of GREL cells, which arise from the mesonephric surface epithelium through proliferation in a process that is also associated with degradation of the basal lamina allowing the primordial germ cells to then associate with GREL cells. Kenngott et al (135) has also observed that the mesonephric surface epithelium was single-layered, except where the gonadal thickening occurs. The stroma, with its leading edge basal lamina, does not penetrate into the ovarian primordium until later in development. A population of GREL cells on the surface eventually develops an epithelial phenotype only after the stroma has expanded to just below the superficial GREL cells (31).

While also observing that the early ovarian primordium does not have a defined surface epithelium, the exception was “at the base where it arises and protrudes from the mesonephros” (31). The base or hilum of the ovary is in fact a protrusion of the mesonephros and is covered by an established classical surface epithelium with a subepithelial basal lamina and epithelial-stromal interface and is derived directly from the mesonephros. The remainder of the ovary has surface epithelial cells initially derived from GREL cells. This could be important because the surface epithelium of the adult mouse ovary is not uniform (136, 137). The hilum or base of the mouse ovary is richer in stem cells with greater oncogenic potential than cells at other locations on the surface of the ovary (136). It is possible, notwithstanding species differences, that the different developmental history of the epithelial cells at the base vs the rest of the ovary contributes to the different behavior of the epithelial cells from different locations in the ovary.

During every ovulation, the ovarian surface is ruptured, and the continuous layer of surface epithelium and the underlying tunica albuginea are damaged. It is assumed that stem cells in the remaining surface epithelium start to proliferate and differentiate to restore the damaged surface. Until recently, little was known about the surface epithelial stem cells (128, 136, 137). The first study to identify possible stem/progenitor cells in the ovarian surface epithelium was performed by Szotek et al (138) in mice. They performed pulse-chase experiments with 5-bromodeoxyuridine and used transgenic mice, which expressed histone 2B-GFP in the presence of doxycycline. They were able to identify a population of long-term label-retaining cells in the surface epithelial layer. These cells were quiescent before ovulation and started replicating near the edges of the ruptured follicle wall after ovulation, indicating that these cells were involved in the remodeling process. Recently, Usongo and Farookhi (139) suggested an involvement of the WNT/β-catenin-signaling in the establishment of a progenitor cell population in the ovarian surface epithelium. The transgenic mice used in the study carried a β-catenin/T-cell factor (TCF)-responsive lacZ reporter gene, thus identifying WNT-activated cells. Interestingly, the lacZ expression occurred in cells of the mediolateral lining of the undifferentiated gonad, whereas after sex determination it was restricted to the female gonad. This is in line with the membranous localization of β-catenin in E12.5 mouse gonads (140). Furthermore, this expression in cells of the ovarian surface epithelium showed an age-dependent decline after birth to a population of 0.2% of the surface epithelial cells. This decline did not result from apoptosis or reduced proliferation, but rather from lacZ-positive cells differentiating into lacZ-negative cells. This suggests that lacZ-positive cells (active β-catenin/TCF signaling) in the ovarian surface epithelium act as stem cells and regenerate the ovarian surface. In a subsequent study, Usongo et al (141) showed the membranous expression of β-catenin in the ovarian surface cells of postnatal mice ovaries. Primary surface epithelial cultures using WNT agonists resulted in increased proliferation and stabilization of β-catenin but did not induce β-catenin/TCF-related transcriptional activity. Recently, it has been shown that WNT4 and RSPO1 up-regulate the adult stem cell marker LGR5 in developing mouse ovaries, again suggesting that this pathway is critical for stem cells of the ovarian surface (128).

Gamwell et al (142) isolated a population of cells with stem cell properties by flow cytometry from the ovarian surface epithelium of adult mice and found that these cells express higher levels of mRNA for the hematopoietic stem cell marker lymphocyte antigen 6 complex, locus A (LY6A). The LY6A^+^ side population, which represents 2% of the total surface epithelial cell population, started proliferating after 4 weeks, whereas LY6A^−^ cells proliferated in the first 7 days of culture. The rate of spheroid formation, a criterion for stem cell properties, was higher in the LY6A^+^ population compared to the other surface epithelial cells. LY6A^+^ cells, as shown by immunohistochemistry, existed in the surface layer and were not in contact with any ovarian structures such as follicle walls or corpora lutea. Furthermore, the cells appeared more cuboidal compared to the remaining surface epithelial cells, and additionally, the oocytes of primordial follicles stained positive for LY6A. Since there is increasing evidence for the existence of germline stem cells on the surface of mouse ovaries (36, 61), it cannot be excluded that the LY6A^+^ cells detected in ovarian tissue sections might be germline stem cells instead of progenitors/stem cells of ovarian surface epithelium. Flesken-Nikitin et al (136) identified cells in the hilum region of postnatal mice ovaries that showed typical stem cell properties such as the expression of ALDH1 (aldehyde dehydrogenase 1), LGR5, CD133 (cluster of differentiation 133), CK6B (cytokeratin 6B), and LEF1 (lymphoid enhancer-binding factor 1), as well as long-term survival/proliferation and spheroid formation in culture. Furthermore, these cells were activated after ovulation to repair the surface epithelium as shown in pulse-chase experiments with 5-bromodeoxyuridine labeling. Interestingly, in *Trp53*- and *Rb1*-deficient mice ovaries, cells in the hilum region appeared to have tumorigenic properties. A further study of LGR5 populations in adult mice by Ng et al (137) identified the embryonic and neonatal LGR5-positive cells as adult stem/progenitor cells in the ovarian surface that are involved in wound repair after ovulation by sealing the damaged ovarian surface. LGR5 expression was located on the surface and subsurface region in the fetal mouse ovary but became restricted to the surface epithelium at postnatal day 7 and in adult mice. Unlike Flesken-Nikitin et al (136), two other studies (128, 137) also observed LGR5-positive cells not only at the hilum of the adult mice ovary (Figure 5) but also throughout the ovarian surface epithelium, in particular at the periphery of rupturing follicles or covering corpora lutea (137). A study in human adult ovaries showed that 75 to 100% of the ovarian surface epithelial cells expressed the known stem cell marker NANOG, secreted frizzled related protein 1 (SFRP1), LIM homeobox 9 (LHX9), and ALDH1A2, but only 25% were positive for ALDH1A1 (143).

In summary, there are two origins of ovarian surface epithelial cells. Most of the initial surface epithelial cells covering the major portion of the fetal ovary are derived from the GREL cells, which originally were derived from the surface epithelial cells of the mesonephros. The hilum of the ovary is directly derived from mesonephros, and its epithelium is a classic epithelium and remains as such while the remainder of the ovary develops. What happens later in life with the movement of cells around the surface is an interesting topic that needs further studies and extension to other species. The stem cell characteristic and oncogenic potential of the epithelial cells are also major issues to be researched further.

## IV. Folliculogenesis

### A. Cells of the thecal layers

The origin(s) of the theca interna and externa has received scant attention (see review in Ref. 144). The thecal layers are first identifiable around the time of antrum formation. The theca interna contains the steroidogenic cells, fibroblasts, immune cells, and capillaries, whereas the externa has larger venules, lymphatic vessels, nerve fibers, and cells with contractile filaments. Earlier studies have, not surprisingly, suggested that thecal cells are recruited from the stroma. However, one study claimed that there is a population of thecal stem cells in mouse ovaries (145). In that study, cells were cultured from whole mouse ovaries, not isolated thecal layers. It was shown that the colonies of cells in soft agar expressed genes known to be expressed by thecal cells (nonquantitative RT-PCR showing *Ptch1* and *2*, *Gli 2* and *3*), but the authors did not confirm whether all the cells or only a proportion of the cells in the colonies expressed these genes (145). These colonies were of mixed cell types, as “many oocytes continued to protrude from the surface of the colonies” (145). Many of the features of these stem cells are features published previously for granulosa stem cells (see *Section IV.B*), such as anchorage-independent growth and their responses to basic fibroblast growth factor (bFGF) and IGF-1. The colonies contained basement membrane material, as granulosa stem cell colonies do (146) and as granulosa cells make (147). Thus, at this time, it is clear that further research is needed to establish whether these mouse cells (145) are indeed thecal stem cells.

Recent evidence suggests that a putative stromal stem cell niche exists in the ovary. Certain chondroitin/dermatan sulfate epitopes (antibodies 7D4, 3C5, and 4C3) have been detected in the ovary (148), which in other tissues marked stem cell niches. The identity of these motifs and indeed the proteoglycan associated with them is currently unknown. However, the same chondroitin/dermatan sulfate epitopes in the ovary are located in the stromal connective tissue surrounding early antral bovine follicles and, in unique clusters of cells, surrounding some vascular elements in the theca externa in large antral follicles (148). Whether these areas in the ovary contain progenitors or thecal stem cells remains to be determined.

Another study in pigs identified an alkaline phosphate-positive cell population in the third passage of cultured thecal cells, which were isolated from follicles larger than 4 mm (149). These cells expressed mesenchymal surface markers, such as CD29, CD44, and CD90, and the pluripotency marker SOX2 but were negative for the expression of OCT4 and NANOG. Furthermore, these cells successfully differentiated into osteocytes, adipocytes, and oocyte-like cells under the specific culture conditions, suggesting the multipotent potential of these cells. The oocyte-like cells formed spheroids and expressed pluripotency markers (OCT4, NANOG, SOX2), oocyte-specific markers (DAZL, VASA, STELLA, ZP, GDF9B, SCP3, C-MOS), and FSH receptor (FSHR).

Immune cells reside in close association with theca cells but are precluded from the membrana granulosa and therefore from directly accessing the follicle or contacting the oocyte. The physical barrier provided by granulosa cells and the follicular basal lamina protect the ovum and prevent access to immune cells during follicle development. Both the oocyte and its surrounding zona pellucida have immunogenic molecules foreign to the mother. Studies in rodents show that a majority of the immune cells in the stroma and theca layers are macrophages and neutrophils (150), cells that are implicated in aspects of tissue remodeling associated with follicle development and progression to ovulation or atresia. Studies in human ovaries show similar patterns of macrophage and neutrophil accumulation in developing follicles (44, 45).

### B. Granulosa cells

**Figure 6.**
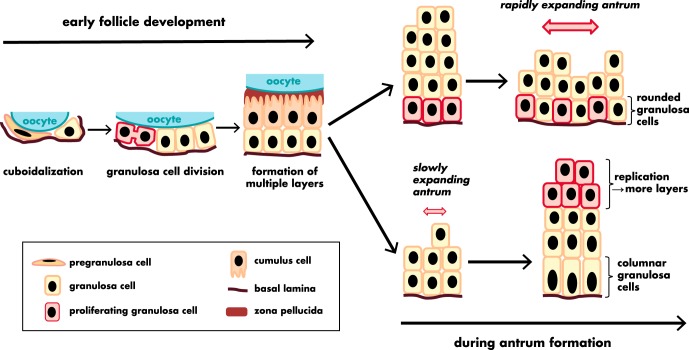
Morphological changes of the granulosa cells and the granulosa layer during follicle development. [Adapted from data derived from P. Da Silva-Buttkus et al: Effect of cell shape and packing density on granulosa cell proliferation and formation of multiple layers during early follicle development in the ovary. *J Cell Sci*. 2008;121:3890–3900 (271) and from R. J. Rodgers and H. F. Irving-Rodgers: Morphological classification of bovine ovarian follicles. *Reproduction*. 2010;139:309–318 (272).]

When first formed, the follicle contains oocytes and GREL/pregranulosa cells. It is not known whether there is a distinction between GREL and pregranulosa cells and, if so, at what stage the transition occurs or what initiates it. However, after growth of the primordial follicle is initiated, proliferation commences in the previously quiescent GREL/pregranulosa cells (Figure 6), and they differentiate into granulosa cells whose function in immature follicles is to support the growth of the oocyte. During growth of the bovine follicles, granulosa cells double in number 21 times from the primordial to antral follicle stage (151). The first evidence that some granulosa cells had properties of stem cells came in 1994 (152) when it was proposed that the membrana granulosa, like other epithelia, is derived from stem cells. It was shown that a proportion of granulosa cells isolated directly from antral follicles has a number of stem cell properties, including the ability to divide under anchorage-independent conditions and form colonies (146, 152–155), divide without contact inhibition (151), and express telomerase (156), with the highest activity in the smaller follicles also supporting a stem cell model as proposed (151). The colonies of granulosa cells produced a basal lamina, and the granulosa cells in colonies could be induced to differentiate into luteal cells with dibutyryl cAMP treatment (152), eliminating the possibility that the colonies might have been derived from contaminating blood cells.

The stem cell model of granulosa cells (151) originally assumed pluripotency, with the ability to differentiate into cumulus and mural granulosa cells. Early research suggested that they exhibited some degree of plasticity because granulosa/cumulus cells were the source of nuclei in successful somatic cell nuclear transfer cloning in cattle (157) and cats (158). Recently, however, the multipotential capacity of granulosa cells has been demonstrated (159–162) and is reviewed in Ref. 163. Luteinizing granulosa cells from follicular aspirates of in vitro fertilization patients were sorted for FSH receptor (FSHR) by FACS and survived long-term culture in the presence of leukemia-inducing factor (LIF) (159). Furthermore, these cells expressed OCT4 and mesenchymal lineage markers such as *CD29*, *CD44*, *CD90*, *CD105*, *CD117*, and *CD166* throughout culture, but gradually they lost *FSHR* and *CYP19A1* expression. Significantly, Kossowska-Tomaszczuk et al (159) were able to differentiate these fibroblast-like stem cells in vitro into neurons, chondrocytes, and osteoblasts under the necessary culture conditions. Another study examined the overall gene expression profile of cells from follicular aspirates of infertile women in comparison to bone marrow-derived mesenchymal stem cells and dermal fibroblasts. Cells in the follicular aspirates expressed mesenchymal stem cell-related markers (*LIF*, *CD106*, *CD146*, *CD45*, *IL10*, *TNF*, *vWF*) and adipogenesis-related, osteogenesis-related (*RUNX*) and chondrogenesis-related (*SOX9*) genes (161). Furthermore, cells from these antral follicles could be differentiated into adipogenic, osteogenic, and pancreatic cells in vitro. However, in this latter study, no attempt was made to isolate granulosa cells from any potential contaminating cells in the aspirates, which would be needed to ensure that it was the granulosa cells that had exhibited the pluripotential in this study.

In addition to mesenchymal stem cell potential, two studies have identified subpopulations with endothelial-like character in human, murine (162), and bovine granulosa cells (160). In the first study, most cultured human cumulus and mural granulosa cells from in vitro fertilization patients and murine granulosa cells expressed endothelial-like markers such as TIE (tyrosine kinase with Ig-like and epidermal growth factor [EGF]-like domains), TEK (endothelial-specific receptor tyrosine kinase), von Willebrand factor, cKIT, CD31, and FLT-1 (VEGF receptor 1), and showed the ability to take up acetylated low-density lipoprotein (162). It was suggested that these cells might be involved in the vascularization process after ovulation because formation of tubular networks was observed in the cultures. In contrast, Merkwitz et al (160) identified somatic progenitor cell colonies that formed on top of monolayer cultures of bovine granulosa cells from antral follicles and had the potential to differentiate into macrophages or endothelial or granulosa cells. These colonies expressed the pluripotency markers SOX-2, OCT-3/4, and cKIT. Most of the cells differentiated into macrophages, but a minority showed coexpression of cKIT and endothelial markers such as CD14, CD45, CD133, and VEGF-R2. Magnetic bead selection of these double-positive cells and subsequent culture in hanging drops in hematopoietic-differentiation medium resulted in pure microspheroids of either endothelial cells or granulosa cells.

Recently, Lan et al (164) successfully differentiated human embryonic stem cells into granulosa-like cells in vitro by transient cell enrichment using different growth factors, bFGF, activin A, BMP4, wingless-type mouse mammary tumor virus integration site family member 3A (WNT3A), and follistatin. These granulosa-like cells expressed *FOXL2*, *CYP19A1*, *FSHR*, *AMH* (anti-Müllerian hormone), and *AMHR2* and were able to produce AMH and convert T into estradiol.

Collectively, these studies indicate that a population of stem cell-like or transit-amplifying cells residing in the granulosa layers may contribute to the high proliferative potential of the ovarian follicle. Extrapolating these findings to smaller follicles, it seems likely that these cells are the direct descendants of the squamous pregranulosa layer in primordial follicles whose lineage is now better understood through the characterization of GREL cells.

### C. Cumulus cells

As follicles grow and become FSH responsive, the follicular antrum forms (165) and the granulosa cell compartment differentiate into two mature cell lineages: the mural granulosa cells, which, as their name indicates, line the follicle wall; and the cumulus cells, which remain in contact with the oocyte. These are distinct cell types because they have different responsiveness to extracellular signals, different roles in the follicle, and different fates, but they are believed to share common lineage origins, being the GREL cells and, subsequently, stem-like cells in the granulosa layers. The mural cell lineage participates mainly in the endocrine feedback control of the reproductive cycle, and later mediates the effects of the midcycle LH surge. This endocrine function is critical to the regulation of the changing hormone levels characteristic of each menstrual cycle, and it is important in determining the fate of each follicle within a growing cohort through the mechanisms described above. The cells in closest proximity to the oocyte form the cumulus cell lineage controlled by localized signals emanating from the oocyte, including TGFβ family members, GDF9 (166) and BMP15 (167) as well as FGFs, and either FGF8b in rodents (168) or FGF10 in humans (169) and ruminants (170). The signal from oocyte-derived ligands is highly spatially restricted, ensuring that only cells closest to the oocyte retain cumulus specification. Often, granulosa cells adjacent to the antral cavity also exhibit cumulus characteristics (for example, see Refs. 171 and 172), perhaps indicating that the antral fluid does not block dissemination of the oocyte-derived factors. This spatial patterning appears to involve the heparan sulfate binding properties of TGFβ and FGF ligands (173). Thus, heparan sulfate proteoglycans in the follicular extracellular matrix restrict diffusion by sequestering the ligands and may also contribute to the signaling receptor interactions (174). The cumulus cell lineage is not steroidogenic because the steroidogenic enzymes and LH receptor are specifically suppressed through the action of GDF9/BMP15 (175). The cumulus cells continue the important role of supporting the development of the growing oocyte and its eventual acquisition of competence to form an embryo with high developmental potential. Specifically, cumulus cells sense and respond to maternal endocrine and paracrine signals to produce energy, cyclic nucleotides (cAMP and cGMP), and RNA and transfer these to the oocyte via gap junctions (176, 177). These factors regulate oocyte meiotic arrest/resumption through cell cycle-regulating complexes (178–180). The glycolytic enzymes are up-regulated in cumulus cells via GDF9/BMP15 action (168, 181), and cumulus cells efficiently metabolize glucose, thus providing glycolytic products to the oocytes as substrates for energy production via oxidative phosphorylation. Recently, it was shown that cumulus cells stimulated by EGF-like signals from the mural granulosa cells directly participate in the control of oocyte mRNA translation by regulating the association of certain RNA transcripts with polysomes (182). Together, these examples demonstrate that cumulus cells perform key functions on behalf of oocytes and act as a nexus by responding to oocyte and maternal stimuli to coordinate oocyte maturation in relation to the reproductive cycle and follicle development (reviewed in Ref. 183).

## V. Ovulation and Corpus Luteum

### A. Cell changes at ovulation

General mechanisms involved in ovulation have been well reviewed (183–185). Granulosa cells express LH receptors and respond to the LH surge by inducing steroidogenic genes involved in luteinization as well as a very rapid and early (within 1 h) production of several EGF-like ligands (186, 187) which act through cognate EGF receptors (EGFR and ERBB2) on both granulosa cells and cumulus cells to stimulate phosphorylation of the ERK signal transducers essential for ovulation (188). The orphan nuclear receptor LRH1 is required for successful ovulation because it permits the normal expression of steroidogenic genes in granulosa as well as cumulus matrix genes. Mice with a granulosa-specific *Lrh1* deletion are unable to ovulate (189). Prostaglandin synthesis induced by the LH surge is also a critical step because a deficiency in the rate-limiting enzyme for the synthesis of cyclooxygenase 2 (COX2) in mice (190) or the injection of COX2 inhibitors in cows (191) led to failure of ovulation. Prostaglandin E2 is involved in cumulus expansion (192), nuclear maturation, and ovulation of mouse (193) and primate (194, 195) oocytes. A transient surge in progesterone receptor expression, peaking around 6 hours after the LH surge, along with the high production of progesterone by differentiating granulosa/lutein cells, has been shown to be essential for ovulation but not luteinization. Ablation of the *Pgr* gene in knockout mouse models revealed that LH-mediated induction specifically of the progesterone receptor isoform A in granulosa cells is essential for ovulation (196, 197). Progesterone antagonist treatments have substantiated this conclusion in many species, including humans (198, 199), ruminants (200), and rodents (196, 201).

At ovulation, the follicle wall at the surface of the ovary is degraded and ruptures at the follicle apex, releasing the cumulus-oocyte complex, and the follicular basal lamina, focimatrix, and the follicle wall at the surface of the ovary are degraded (202). Interestingly, the basal laminas of the nearby thecal capillaries are preserved (202), suggesting that the degradation of the extracellular matrix at ovulation must involve a degree of precision, but how this precision is achieved is currently not known. A number of extracellular matrix proteases are expressed in ovulating follicles. Two progesterone receptor-mediated proteases, cathepsin-L and ADAMTS1 (ADAM metallopeptidase with thrombospondin motif 1), were expected to be important because of the absolute influence of the progesterone receptor on ovulation. Indeed, studies of *Adamts1* null mice confirmed that this is an important mediator of ovulation (203). Several roles for ADAMTS1 include the promotion of lymphangiogenesis (88, 204) and the degradation of its best known substrate, versican, and possibly other extracellular matrix components in the follicle wall, as well as in the cumulus-oocyte complex matrix (203, 205, 206). This occurs immediately around the time of ovulation (203, 205, 206). Metalloproteinase inhibition has been shown to prevent rupture of the follicle surface in a number of species including primates (207). Several members of the membrane-associated MMPs (matrix metalloproteinases), which activate extracellular proteases, are also increased in granulosa cells after the LH surge (208).

Striking morphological changes also occur in the cumulus-oocyte complexes during ovulation in all mammalian species. As already mentioned, cumulus cells do not express LHR, but they respond rapidly after the LH surge through EGF-like ligands produced by granulosa cells. These ligands are cell membrane associated, and evidence suggests that they are rapidly cleaved by proteases possibly including ADAM (a disintegrin and metalloprotease) and ADAMTS. Within the cumulus-oocyte complex, a number of extracellular matrix genes are activated, including *Has2*, which synthesizes hyaluronan (209) along with a complex of hyaluronan cross-linking proteins (210–212) including TNF-α-stimulated protein 6 (*Tnfaip6* [213; reviewed in Ref. 214]) and *Ptx3* (pentraxin 3 [215, 216]). Hyaluronan is then bound by versican, which is an abundant product of granulosa cells, and diffuses to the cumulus-oocyte complex (217–219). Heavy chain of inter-α-inhibitor enters the follicle from the blood stream due to elevated vascular permeability and breakdown of the basal lamina and binds the hyaluronan cross-linking protein complex (220–223). This unique cumulus-oocyte complex matrix composition is critical for the success of ovulation. Disruptions to any of the above-mentioned genes markedly reduces the ovulation rate (183). How the matrix of the cumulus-oocyte complex promotes ovulation remains to be demonstrated, but it has been shown that cumulus-oocyte complex expansion is associated with adhesion to extracellular matrix (collagen I, III, and IV, as well as fibronectin) and strong invasive capacity of cumulus cells (224), which are likely to be keys to detachment from the granulosa layers, release of the cumulus-oocyte complex from the follicle, and binding to the oviduct or fallopian tube lining.

The rupture of the follicle wall is accompanied by an inflammation-like process (reviewed in Ref. 225) with up-regulation in leukocytes and nonhematopoietic cells of the follicle of cytokines including IL-1α (226, 227), IL-1β (228), IL-4 (226, 227), and IL-6 (229) and prostaglandins (230). Induction of the angiogenic growth factor VEGFα in granulosa cells through the actions of LH in cooperation with hypoxia (231) activates angiogenesis in the vasculature that surrounds ovulating follicles, leading to the migration and infiltration of endothelial cells into the follicle interior. Recruitment of immune cells into the thecal layers (43) occurs, and leukocytes invade the granulosa cell layers. Influx of inflammatory cells including mast cells, T cells, neutrophils, and macrophages into the tissue surrounding ovulating follicles plays an important role in the remodeling processes. Depletion of macrophages using clodronate liposomes (232) or genetic deficiency in the macrophage-regulating cytokine colony stimulating factor 1 (233) supports the concept that these cells are crucial for follicle maturation and ovulation. One key role is secretion of proteases that degrade extracellular matrix proteins and cause the follicle wall to weaken and eventually to rupture under progressively elevated follicular edema. Most recently, dendritic cells were shown to be required for ovulation (234), potentially through roles in regulating the inflammatory status of associated cells, such as T cells and macrophages.

After ovulation, the surface epithelium is repaired at the point of rupture. Some surface epithelial cells of the ovary express LGR5, a marker of epithelial stem cells, and although it has not been completely proven, these cells are probably responsible for maintaining the populations of surface epithelial cells on the ovary. Currently there are two proposals explaining how this might occur. One suggestion is that the stem cells reside at the base or hilum of the ovary and act as a reserve of surface epithelial cells for the rest of the ovary (136). Another study confirmed these stem cells at the hilum of the ovary but additionally found pockets of LGR5-positive cells dispersed around the remainder of the ovary (128) and increasingly near ovulation points (137). Additional investigations are needed to extend these findings to other species.

### B. Cells of the corpus luteum

After ovulation, the remaining cells and matrices of the follicle wall undergo remodeling leading to the formation of the corpus luteum. The structural and functional changes involved with the formation and regression of the corpus luteum have been extensively reviewed (235). The basal lamina separating the membrana granulosa from the theca interna loses its integrity, allowing cells from both layers to intermingle. Capillaries, previously confined to the thecal layers, penetrate the granulosa cell layers and the follicular antrum and subsequently vascularize the developing corpus luteum. The follicular extracellular matrix is completely remodeled during the follicular-luteal transition (202, 236) (reviewed in Ref. 237), and the composition of the extracellular matrix of the corpus luteum has been described for human (238), cow, sheep (239), mouse (240), and rat (241) (reviewed in Ref. 242). The resultant corpus luteum is therefore vascularized mesenchymal tissue rather than a stratified epithelium, and the granulosa cells can be described as undergoing an epithelial-mesenchymal transition (147, 243–245) differentiating into granulosa lutein (primates) or large luteal (other species) cells. Cellular luteinization, which can be easily modeled in culture (246), is characterized by hypertrophy, a greatly increased capacity for progesterone synthesis, and altered patterns of peptide and protein secretion. Luteinization in vitro appears to be promoted by exposing the cells to extracellular matrix, and the luteinizing cells themselves contribute to the deposition and remodeling of matrix material (247–250). The steroidogenic cells of the theca interna develop into the theca lutein or small luteal cells. In humans, the latter remain located in the periphery of the corpus luteum, whereas in ruminants the small luteal cells intersperse between the large luteal cells (251, 252). In rodents, it is still not clear whether thecal-derived luteal cells exist.

Immune cells including macrophages, neutrophils, and dendritic cells are abundant in the developing corpus luteum (44, 45, 253, 254) and are thought to facilitate tissue remodeling events as well as control of steroidogenic function (255–258). Recently, macrophages have been shown to be crucial for the development and maintenance of the corpus luteum. Mice with macrophage depletion showed disruptions in the luteal vascular network caused by altered gene expression for VEGFs and increased expression of genes related to inflammation and apoptosis. Furthermore, essential genes involved in progesterone synthesis, such as for steroidogenic acute regulatory protein, cytochrome P450 cholesterol side-chain cleavage, and 3β-hydroxysteroid dehydrogenase, were diminished, leading to failure of implantation in these mice (259). The key role of macrophages was shown to be through production of VEGFs that facilitate the rapid neovascularization crucial for corpus luteum development and timely production and secretion of progesterone to enable progression of pregnancy.

Macrophages are not the only important immune cells in the corpus luteum. Notably, studies in bovine corpus luteum reveal a complex array of T cells, with a profile of phenotypes that fluctuates over the course of the luteal lifespan, implying local environmental control of T-cell populations. The T-cell pool in the corpus luteum has a different composition with enrichment of CD8^+^ cells compared to blood, and many CD4^+^ and CD8^+^ Treg cells, which presumably have an immune suppressive phenotype, are widely prevalent (260).

If fertilization and progression to pregnancy do not occur, the corpus luteum will undergo luteolysis to allow development toward ovulation of a new antral follicle. The luteolytic process involves structural and functional changes. In primates, the regulation of luteolysis is still not clearly understood but involves chorionic gonadotropin “rescuing” the corpus luteum from demise (261). In ruminants, luteolysis is mainly mediated by prostaglandin F2α (PGF2α) secreted by the endometrium (reviewed in Refs. 262 and 263). Injections of luteolytic doses of PGF2α caused a decrease in superoxide dismutase after 24 hours in bovine corpora lutea in vivo and in cultured luteal endothelial cells (264). A decline in antioxidants and antioxidative enzymes results in an increase of reactive oxygen species and reactive oxygen species-associated apoptosis of the cells of the corpus luteum. PGF2α decreases the expression of genes and proteins, such as low-density lipoprotein receptor, steroidogenic acute regulatory protein, or 3β-hydroxysteroid dehydrogenase, which are involved in progesterone biosynthesis in porcine corpora lutea (265). This results in a decrease in progesterone production. On the other hand, PGF2α significantly increases the expression of members of the SLIT/Roundabout (ROBO) receptor signaling, *Slit2* and *Robo1*, through activation of protein kinase C-dependent ERK1/2 and MAPK signaling pathways during luteolysis in mice (266). Finally, this results in the up-regulation of cleaved caspase-3 and apoptosis. In the human corpus luteum, *SLIT2* and *ROBO1* as well as *SLIT2*, *SLIT3*, *ROBO1*, and *ROBO2* have been shown to be expressed the highest in the steroidogenic cells and fibroblast-like cells of the late luteal phase (267), but how these are connected to luteolysis is not clear.

Immune cells also contribute to the demise of corpora lutea. Importantly, a sharp 10-fold decline in CD4^+^CD25^+^ FOXP3^+^ Treg cells, accompanied by reduced *TGFB1* expression, is observed 8 hours after luteolysis triggered by PGF2α administration (260). This decline in Treg cells may be a direct cause of the shift toward a proinflammatory state, which in turn precedes progesterone decline (268). Sudden loss of Treg-mediated suppression would be expected to elevate secretion of the proinflammatory cytokines known to inhibit progesterone secretion (256, 269) and may accelerate apoptosis-mediated cell death and structural demise. This is consistent with evidence that a proinflammatory insult terminates corpora lutea function through an immune mechanism (270).

## VI. Conclusions and Perspectives

The cellular biology of the ovary and its follicles and corpora lutea is critical to the endocrine and fertility functions of the ovary, and yet we still have much to discover. As with all new fields, many concepts that are investigated will not be fruitful. We believe that the evidence of thecal stem cells, VSEL cells, and oogonial cells arising from the bone marrow is not convincing at this stage. In adult ovaries, germline stem cells have been proposed to form oocytes and contribute to the formation of new follicles, but the evidence for this occurring in vivo is also lacking.

A new model on how the ovary develops challenges and expands some previous concepts on the origins of granulosa cells and even the surface epithelial cells. Both have been suggested to be derived from GREL cells. The surface epithelium appears more complex, with cells derived from GREL cells in the lateral and apical regions and directly from the mesonephric surface at the hilum of the ovary. The formation of the ovigerous cords appears to be due to the action of the stroma branching as it penetrates into the ovary from the mesonephros, and indeed its action appears to be important in both follicle formation from ovigerous cords and in the formation of the ovarian surface epithelium. Many questions now arise. What controls the activity of the stroma during these early developmental phases? What causes the stroma that penetrates to below the surface epithelium to develop into the tunica albuginea? What initiates the formation of GREL cells? What changes do GREL cells undergo to become surface epithelial cells, and what role do the underlying basal lamina and stroma play in this process? Do the oocytes influence GREL cells to become granulosa cells, and what factors might be involved?

In adult ovaries, evidence for granulosa stem cells has accumulated over 20 years and is convincing and logical as other epithelia operate on a similar model. Evidence now suggests that the ovarian surface epithelium in adult ovaries also acts like other epithelia with stem cells. Are the different developmental origins of surface epithelial cells involved to different degrees in the development of ovarian cancers? Immune cells have now been shown to have critical roles in the ovary also, not just in protecting the oocyte but in critical functions like ovulation and formation, function, and regression of the corpus luteum. The identification of female germline (oogonial) stem cells is still very new and is currently limited to just a few species. Their origins and roles in vivo, if any, are yet to be elucidated. Even if they do not have any roles in vivo, our ability to manipulate them and harness their potential offers many exciting opportunities. Clearly, the future will bring many more significant discoveries.

## References

[1] Hsueh AJ Kawamura K Cheng Y Fauser BC Intraovarian control of early folliculogenesis [published online September 9, 2014]. Endocr Rev. doi.org/10.1210/er.2014–1020. 10.1210/er.2014-1020 PMC4309737 25202833

[2] Matsuda F Inoue N Manabe N Ohkura S Follicular growth and atresia in mammalian ovaries: regulation by survival and death of granulosa cells. J Reprod Dev. 2012;58:44–50. 22450284 10.1262/jrd.2011-012

[3] Scaramuzzi RJ Baird DT Campbell BK Regulation of folliculogenesis and the determination of ovulation rate in ruminants. Reprod Fertil Dev. 2011;23:444–467. 21426863 10.1071/RD09161

[4] Chaves RN de Matos MH Buratini J Jr de Figueiredo JR The fibroblast growth factor family: involvement in the regulation of folliculogenesis. Reprod Fertil Dev. 2012;24:905–915. 22935151 10.1071/RD11318

[5] Chaves RN Alves AM Lima LF Matos HM Rodrigues AP Figueiredo JR Role of nerve growth factor (NGF) and its receptors in folliculogenesis. Zygote. 2013;21:187–197. 22651979 10.1017/S0967199412000111

[6] Inoue N Matsuda F Goto Y Manabe N Role of cell-death ligand-receptor system of granulosa cells in selective follicular atresia in porcine ovary. J Reprod Dev. 2011;57:169–175. 21551974 10.1262/jrd.10-198e

[7] Bland ML Desclozeaux M Ingraham HA Tissue growth and remodeling of the embryonic and adult adrenal gland. Ann NY Acad Sci. 2003;995:59–72. 12814939 10.1111/j.1749-6632.2003.tb03210.x

[8] Mitchell RT Saunders PT Childs AJ Xenografting of human fetal testis tissue: a new approach to study fetal testis development and germ cell differentiation. Hum Reprod. 2010;25:2405–2414. 20683063 10.1093/humrep/deq183 PMC2939754

[9] Robin C Ottersbach K de Bruijn M Ma X van der Horn K Dzierzak E Developmental origins of hematopoietic stem cells. Oncol Res. 2003;13:315–321. 12725520 10.3727/096504003108748519

[10] Thullberg M Strömblad S Anchorage-independent cytokinesis as part of oncogenic transformation? Cell Cycle. 2008;7:984–988. 18414025 10.4161/cc.7.8.5674

[11] Nelson PJ Daniel TO Emerging targets: molecular mechanisms of cell contact-mediated growth control. Kidney Int. 2002;61:S99–S105. 11841621 10.1046/j.1523-1755.2002.0610s1099.x

[12] Potten CS Owen G Booth D Intestinal stem cells protect their genome by selective segregation of template DNA strands. J Cell Sci. 2002;115:2381–2388. 12006622 10.1242/jcs.115.11.2381

[13] Shinin V Gayraud-Morel B Gomès D Tajbakhsh S Asymmetric division and cosegregation of template DNA strands in adult muscle satellite cells. Nat Cell Biol. 2006;8:677–687. 16799552 10.1038/ncb1425

[14] Bickenbach JR Vormwald-Dogan V Bachor C Bleuel K Schnapp G Boukamp P Telomerase is not an epidermal stem cell marker and is downregulated by calcium. J Invest Dermatol. 1998;111:1045–1052. 9856815 10.1046/j.1523-1747.1998.00420.x

[15] Lane AA Scadden DT Stem cells and DNA damage: persist or perish? Cell. 2010;142:360–362. 20691895 10.1016/j.cell.2010.07.030

[16] Milyavsky M Gan OI Trottier M A distinctive DNA damage response in human hematopoietic stem cells reveals an apoptosis-independent role for p53 in self-renewal. Cell Stem Cell. 2010;7:186–197. 20619763 10.1016/j.stem.2010.05.016

[17] Mohrin M Bourke E Alexander D Hematopoietic stem cell quiescence promotes error-prone DNA repair and mutagenesis. Cell Stem Cell. 2010;7:174–185. 20619762 10.1016/j.stem.2010.06.014 PMC2924905

[18] Møllgård K Jespersen A Lutterodt MC Yding Andersen C Høyer PE Byskov AG Human primordial germ cells migrate along nerve fibers and Schwann cells from the dorsal hind gut mesentery to the gonadal ridge. Mol Hum Reprod. 2010;16:621–631. 20566702 10.1093/molehr/gaq052

[19] Maheshwari A Fowler PA Primordial follicular assembly in humans–revisited. Zygote. 2008;16:285–296. 18616843 10.1017/S0967199408004802

[20] Wilhelm D Palmer S Koopman P Sex determination and gonadal development in mammals. Physiol Rev. 2007;87:1–28. 17237341 10.1152/physrev.00009.2006

[21] Bendel-Stenzel M Anderson R Heasman J Wylie C The origin and migration of primordial germ cells in the mouse. Semin Cell Dev Biol. 1998;9:393–400. 9813186 10.1006/scdb.1998.0204

[22] Chuva de Sousa Lopes SM Roelen BA On the formation of germ cells: the good, the bad and the ugly. Differentiation. 2010;79:131–140. 20227006 10.1016/j.diff.2009.11.003

[23] Matsui Y The molecular mechanisms regulating germ cell development and potential. J Androl. 2010;31:61–65. 19875497 10.2164/jandrol.109.008094

[24] Saitou M Yamaji M Germ cell specification in mice: signaling, transcription regulation, and epigenetic consequences. Reproduction. 2010;139:931–942. 20371640 10.1530/REP-10-0043

[25] De Felici M Origin, migration, and proliferation of human primordial germ cells. In: Coticchio G Albertini DF De Santis L, eds. Oogenesis. London, UK: Springer; 2013:19–37.

[26] Eggers S Sinclair A Mammalian sex determination—insights from humans and mice. Chromosome Res. 2012;20:215–238. 22290220 10.1007/s10577-012-9274-3 PMC3279640

[27] Bonilla-Musoles F Renau J Hernandez-Yago J Torres J How do oocytes disappear? Arch Gynakol. 1975;218:233–241. 1174310 10.1007/BF00667384

[28] Motta PM Makabe S Elimination of germ cells during differentiation of the human ovary: an electron microscopic study. Eur J Obstet Gynecol Reprod Biol. 1986;22:271–286. 3770277 10.1016/0028-2243(86)90115-2

[29] Motta PM Makabe S Germ cells in the ovarian surface during fetal development in humans. A three-dimensional microanatomical study by scanning and transmission electron microscopy. J Submicrosc Cytol. 1986;18:271–290. 3712511

[30] Kerr JB Duckett R Myers M Britt KL Mladenovska T Findlay JK Quantification of healthy follicles in the neonatal and adult mouse ovary: evidence for maintenance of primordial follicle supply. Reproduction. 2006;132:95–109. 16816336 10.1530/rep.1.01128

[31] Hummitzsch K Irving-Rodgers HF Hatzirodos N A new model of development of the mammalian ovary and follicles. PloS One. 2013;8:e55578. 23409002 10.1371/journal.pone.0055578 PMC3567121

[32] Zou K Yuan Z Yang Z Production of offspring from a germline stem cell line derived from neonatal ovaries. Nat Cell Biol. 2009;11:631–636. 19363485 10.1038/ncb1869

[33] White YA Woods DC Takai Y Ishihara O Seki H Tilly JL Oocyte formation by mitotically active germ cells purified from ovaries of reproductive-age women. Nat Med. 2012;18:413–421. 22366948 10.1038/nm.2669 PMC3296965

[34] Zuckerman S The number of oocytes in the mature ovary. Recent Prog Horm Res. 1951;6:63–109.

[35] Johnson J Canning J Kaneko T Pru JK Tilly JL Germline stem cells and follicular renewal in the postnatal mammalian ovary. Nature. 2004;428:145–150. 15014492 10.1038/nature02316

[36] Zhang Y Yang Z Yang Y Production of transgenic mice by random recombination of targeted genes in female germline stem cells. J Mol Cell Biol. 2011;3:132–141. 21149239 10.1093/jmcb/mjq043

[37] Zhou L Wang L Kang JX Production of fat-1 transgenic rats using a post-natal female germline stem cell line. Mol Hum Reprod. 2014;20:271–281. 24258451 10.1093/molehr/gat081

[38] Johnson J Bagley J Skaznik-Wikiel M Oocyte generation in adult mammalian ovaries by putative germ cells in bone marrow and peripheral blood. Cell. 2005;122:303–315. 16051153 10.1016/j.cell.2005.06.031 PMC11771209

[39] Eggan K Jurga S Gosden R Min IM Wagers AJ Ovulated oocytes in adult mice derive from non-circulating germ cells. Nature. 2006;441:1109–1114. 16799565 10.1038/nature04929

[40] Lee HJ Selesniemi K Niikura Y Bone marrow transplantation generates immature oocytes and rescues long-term fertility in a preclinical mouse model of chemotherapy-induced premature ovarian failure. J Clin Oncol. 2007;25:3198–3204. 17664466 10.1200/JCO.2006.10.3028

[41] Notarianni E Reinterpretation of evidence advanced for neo-oogenesis in mammals, in terms of a finite oocyte reserve. J Ovarian Res. 2011;4:1. 21211009 10.1186/1757-2215-4-1 PMC3024995

[42] Zulli A Rai S Buxton BF Burrell LM Hare DL Co-localization of angiotensin-converting enzyme 2-, octomer-4- and CD34-positive cells in rabbit atherosclerotic plaques. Exp Physiol. 2008;93:564–569. 18192339 10.1113/expphysiol.2007.040204 PMC7197899

[43] Brännström M Mayrhofer G Robertson SA Localization of leukocyte subsets in the rat ovary during the periovulatory period. Biol Reprod. 1993;48:277–286. 8439617 10.1095/biolreprod48.2.277

[44] Brannstrom M Pascoe V Norman RJ McClure N Localization of leukocyte subsets in the follicle wall and in the corpus luteum throughout the human menstrual cycle. Fertil Steril. 1994;61:488–495. 8137972

[45] Best CL Pudney J Welch WR Burger N Hill JA Localization and characterization of white blood cell populations within the human ovary throughout the menstrual cycle and menopause. Hum Reprod. 1996;11:790–797. 8671330 10.1093/oxfordjournals.humrep.a019256

[46] Samy ET Parker LA Sharp CP Tung KS Continuous control of autoimmune disease by antigen-dependent polyclonal CD4+CD25+ regulatory T cells in the regional lymph node. J Exp Med. 2005;202:771–781. 16172257 10.1084/jem.20041033 PMC2212949

[47] Samy ET Setiady YY Ohno K Pramoonjago P Sharp C Tung KS The role of physiological self-antigen in the acquisition and maintenance of regulatory T-cell function. Immunol Rev. 2006;212:170–184. 16903914 10.1111/j.0105-2896.2006.00404.x

[48] Alard P Thompson C Agersborg SS Endogenous oocyte antigens are required for rapid induction and progression of autoimmune ovarian disease following day-3 thymectomy. J Immunol. 2001;166:4363–4369. 11254690 10.4049/jimmunol.166.7.4363

[49] Reizel Y Itzkovitz S Adar R Cell lineage analysis of the mammalian female germline. PLoS Genet. 2012;8:e1002477. 22383887 10.1371/journal.pgen.1002477 PMC3285577

[50] Henderson SA Edwards RG Chiasma frequency and maternal age in mammals. Nature. 1968;218:22–28. 4230650 10.1038/218022a0

[51] Pacchiarotti J Maki C Ramos T Differentiation potential of germ line stem cells derived from the postnatal mouse ovary. Differentiation. 2010;79:159–170. 20138422 10.1016/j.diff.2010.01.001

[52] Zhang Y Wu J Molecular cloning and characterization of a new gene, *Oocyte-G1*. J Cell Physiol. 2009;218:75–83. 18727094 10.1002/jcp.21569

[53] Imudia AN Wang N Tanaka Y White YA Woods DC Tilly JL Comparative gene expression profiling of adult mouse ovary-derived oogonial stem cells supports a distinct cellular identity. Fertil Steril. 2013;100:1451–1458. 23876535 10.1016/j.fertnstert.2013.06.036 PMC4270279

[54] Park ES Woods DC Tilly JL Bone morphogenetic protein 4 promotes mammalian oogonial stem cell differentiation via Smad1/5/8 signaling. Fertil Steril. 2013;100:1468–1475. 23993924 10.1016/j.fertnstert.2013.07.1978 PMC4266321

[55] Lawson KA Dunn NR Roelen BA Bmp4 is required for the generation of primordial germ cells in the mouse embryo. Genes Dev. 1999;13:424–436. 10049358 10.1101/gad.13.4.424 PMC316469

[56] Childs AJ Kinnell HL Collins CS BMP signaling in the human fetal ovary is developmentally regulated and promotes primordial germ cell apoptosis. Stem Cells. 2010;28:1368–1378. 20506112 10.1002/stem.440 PMC2964513

[57] Le Bouffant R Souquet B Duval N Msx1 and Msx2 promote meiosis initiation. Development. 2011;138:5393–5402. 22071108 10.1242/dev.068452

[58] Abban G Johnson J Stem cell support of oogenesis in the human. Hum Reprod. 2009;24:2974–2978. 19687054 10.1093/humrep/dep281

[59] Castrillon DH Quade BJ Wang TY Quigley C Crum CP The human VASA gene is specifically expressed in the germ cell lineage. Proc Natl Acad Sci USA. 2000;97:9585–9590. 10920202 10.1073/pnas.160274797 PMC16908

[60] Lange UC Saitou M Western PS Barton SC Surani MA The fragilis interferon-inducible gene family of transmembrane proteins is associated with germ cell specification in mice. BMC Dev Biol. 2003;3:1. 12659663 10.1186/1471-213X-3-1 PMC153542

[61] Zou K Hou L Sun K Xie W Wu J Improved efficiency of female germline stem cell purification using fragilis-based magnetic bead sorting. Stem Cells Dev. 2011;20:2197–2204. 21615296 10.1089/scd.2011.0091

[62] Zhang H Zheng W Shen Y Adhikari D Ueno H Liu K Experimental evidence showing that no mitotically active female germline progenitors exist in postnatal mouse ovaries. Proc Natl Acad Sci USA. 2012;109:12580–12585. 22778414 10.1073/pnas.1206600109 PMC3411966

[63] Park ES Tilly JL Use of DEAD-box polypeptide-4 (Ddx4) gene promoter-driven fluorescent reporter mice to identify mitotically active germ cells in post-natal mouse ovaries. Mol Hum Reprod. 2015;21:58–65. 25147160 10.1093/molehr/gau071 PMC4275040

[64] Lei L Spradling AC Female mice lack adult germ-line stem cells but sustain oogenesis using stable primordial follicles. Proc Natl Acad Sci USA. 2013;110:8585–8590. 23630252 10.1073/pnas.1306189110 PMC3666718

[65] Wang H Jiang M Bi H Conversion of female germline stem cells from neonatal and prepubertal mice into pluripotent stem cells. J Mol Cell Biol. 2014;6:164–171. 24755856 10.1093/jmcb/mju004

[66] Xie W Wang H Wu J Similar morphological and molecular signatures shared by female and male germline stem cells. Sci Rep. 2014;4:5580. 24993338 10.1038/srep05580 PMC4082104

[67] Virant-Klun I Rozman P Cvjeticanin B Parthenogenetic embryo-like structures in the human ovarian surface epithelium cell culture in postmenopausal women with no naturally present follicles and oocytes. Stem Cells Dev. 2009;18:137–149. 18605894 10.1089/scd.2007.0238

[68] Virant-Klun I Skutella T Hren M Isolation of small SSEA-4-positive putative stem cells from the ovarian surface epithelium of adult human ovaries by two different methods. Biomed Res Int. 2013;2013:690415. 23509763 10.1155/2013/690415 PMC3590614

[69] Virant-Klun I Skutella T Kubista M Vogler A Sinkovec J Meden-Vrtovec H Expression of pluripotency and oocyte-related genes in single putative stem cells from human adult ovarian surface epithelium cultured in vitro in the presence of follicular fluid. Biomed Res Int. 2013;2013:861460. 23555100 10.1155/2013/861460 PMC3600261

[70] Virant-Klun I Skutella T Stimpfel M Sinkovec J Ovarian surface epithelium in patients with severe ovarian infertility: a potential source of cells expressing markers of pluripotent/multipotent stem cells. J Biomed Biotechnol. 2011;2011:381928. 22187524 10.1155/2011/381928 PMC3237017

[71] Virant-Klun I Zech N Rozman P Putative stem cells with an embryonic character isolated from the ovarian surface epithelium of women with no naturally present follicles and oocytes. Differentiation. 2008;76:843–856. 18452550 10.1111/j.1432-0436.2008.00268.x

[72] Parte S Bhartiya D Manjramkar DD Chauhan A Joshi A Stimulation of ovarian stem cells by follicle stimulating hormone and basic fibroblast growth factor during cortical tissue culture. J Ovarian Res. 2013;6:20. 23547966 10.1186/1757-2215-6-20 PMC3635909

[73] Bhartiya D Sriraman K Gunjal P Modak H Gonadotropin treatment augments postnatal oogenesis and primordial follicle assembly in adult mouse ovaries? J Ovarian Res. 2012;5:32. 23134576 10.1186/1757-2215-5-32 PMC3616927

[74] Parte S Bhartiya D Telang J Detection, characterization, and spontaneous differentiation in vitro of very small embryonic-like putative stem cells in adult mammalian ovary. Stem Cells Dev. 2011;20:1451–1464. 21291304 10.1089/scd.2010.0461 PMC3148829

[75] Patel H Bhartiya D Parte S Gunjal P Vedulkar S Bhatt M Follicle stimulating hormone modulates ovarian stem cells through alternately spliced receptor variant FSH-R3. J Ovarian Res. 2013;6:52. 23870332 10.1186/1757-2215-6-52 PMC3728228

[76] Bukovsky A Ayala ME Dominguez R Svetlikova M Selleck-White R Bone marrow derived cells and alternative pathways of oogenesis in adult rodents. Cell Cycle. 2007;6:2306–2309. 17890900 10.4161/cc.6.18.4707

[77] Bukovsky A Caudle MR Gupta SK Mammalian neo-oogenesis and expression of meiosis-specific protein SCP3 in adult human and monkey ovaries. Cell Cycle. 2008;7:683–686. 18256545 10.4161/cc.7.5.5453

[78] Bukovsky A Caudle MR Svetlikova M Upadhyaya NB Origin of germ cells and formation of new primary follicles in adult human ovaries. Reprod Biol Endocrinol. 2004;2:20. 15115550 10.1186/1477-7827-2-20 PMC420494

[79] Bukovsky A Svetlikova M Caudle MR Oogenesis in cultures derived from adult human ovaries. Reprod Biol Endocrinol. 2005;3:17. 15871747 10.1186/1477-7827-3-17 PMC1131924

[80] Bukovsky A Caudle MR Immunoregulation of follicular renewal, selection, POF, and menopause in vivo, vs. neo-oogenesis in vitro, POF and ovarian infertility treatment, and a clinical trial. Reprod Biol Endocrinol. 2012;10:97. 23176151 10.1186/1477-7827-10-97 PMC3551781

[81] Rodgers RJ Hummitzsch K New model of formation of the ovary. Robinson Research Institute. http://www.youtube.com/watch?v=1O97DtAyaDc. Published September 12, 2014.

[82] Juengel JL Sawyer HR Smith PR Origins of follicular cells and ontogeny of steroidogenesis in ovine fetal ovaries. Mol Cell Endocrinol. 2002;191:1–10. 12044912 10.1016/s0303-7207(02)00045-x

[83] Kovacic JC Moore J Herbert A Ma D Boehm M Graham RM Endothelial progenitor cells, angioblasts, and angiogenesis–old terms reconsidered from a current perspective. Trends Cardiovasc Med. 2008;18:45–51. 18308194 10.1016/j.tcm.2007.12.002

[84] Brennan J Karl J Capel B Divergent vascular mechanisms downstream of Sry establish the arterial system in the XY gonad. Dev Biol. 2002;244:418–428. 11944948 10.1006/dbio.2002.0578

[85] Coveney D Cool J Oliver T Capel B Four-dimensional analysis of vascularization during primary development of an organ, the gonad. Proc Natl Acad Sci USA. 2008;105:7212–7217. 18480267 10.1073/pnas.0707674105 PMC2438229

[86] McFee RM Cupp AS Vascular contributions to early ovarian development: potential roles of VEGFA isoforms. Reprod Fertil Dev. 2013;25:333–342. 23021322 10.1071/RD12134

[87] Brown HM Dunning KR Robker RL Pritchard M Russell DL Requirement for ADAMTS-1 in extracellular matrix remodeling during ovarian folliculogenesis and lymphangiogenesis. Dev Biol. 2006;300:699–709. 17097630 10.1016/j.ydbio.2006.10.012

[88] Brown HM Robker RL Russell DL Development and hormonal regulation of the ovarian lymphatic vasculature. Endocrinology. 2010;151:5446–5455. 20843998 10.1210/en.2010-0629

[89] Svingen T François M Wilhelm D Koopman P Three-dimensional imaging of Prox1-EGFP transgenic mouse gonads reveals divergent modes of lymphangiogenesis in the testis and ovary. PloS One. 2012;7:e52620. 23285114 10.1371/journal.pone.0052620 PMC3527586

[90] Rutkowski JM Ihm JE Lee ST VEGFR-3 neutralization inhibits ovarian lymphangiogenesis, follicle maturation, and murine pregnancy. Am J Pathol. 2013;183:1596–1607. 24036251 10.1016/j.ajpath.2013.07.031 PMC3814520

[91] Hughesdon PE Morphology and morphogenesis of the Stein-Leventhal ovary and of so-called “hyperthecosis”. Obstet Gynecol Surv. 1982;37:59–77. 7033852 10.1097/00006254-198202000-00001

[92] Stein IF Leventhal ML Amenorhea associated with bilateral polcystic ovaries. Am J Obstet Gynecol. 1935;29:181–191.

[93] Lesnoy SK [Partial ovary resection upon oligomenorrhea and amenorrhea.] Gynecol Obstet. 1928;2:184–191.

[94] Urbanek M Woodroffe A Ewens KG Candidate gene region for polycystic ovary syndrome on chromosome 19p13.2. J Clin Endocrinol Metab. 2005;90:6623–6629. 16091490 10.1210/jc.2005-0622

[95] Hatzirodos N Bayne RA Irving-Rodgers HF Linkage of regulators of TGF-β activity in the fetal ovary to polycystic ovary syndrome. FASEB J. 2011;25:2256–2265. 21411746 10.1096/fj.11-181099 PMC3219214

[96] Chaudhry SS Cain SA Morgan A Dallas SL Shuttleworth CA Kielty CM Fibrillin-1 regulates the bioavailability of TGFβ1. J Cell Biol. 2007;176:355–367. 17242066 10.1083/jcb.200608167 PMC2063961

[97] Christner PJ Ayitey S Extracellular matrix containing mutated fibrillin-1 (Fbn1) down regulates Col1a1, Col1a2, Col3a1, Col5a1, and Col5a2 mRNA levels in Tsk/+ and Tsk/Tsk embryonic fibroblasts. Amino Acids. 2006;30:445–451. 16583319 10.1007/s00726-005-0265-y

[98] Prodoehl MJ Irving-Rodgers HF Bonner W Fibrillins and latent TGFβ binding proteins in bovine ovaries of offspring following high or low protein diets during pregnancy of dams. Mol Cell Endocrinol. 2009;307:133–141. 19524133 10.1016/j.mce.2009.03.002

[99] van Wezel IL Rodgers RJ Morphological characterization of bovine primordial follicles and their environment in vivo. Biol Reprod. 1996;55:1003–1011. 8902210 10.1095/biolreprod55.5.1003

[100] Murdoch WJ Programmed cell death in preovulatory ovine follicles. Biol Reprod. 1995;53:8–12. 7669860 10.1095/biolreprod53.1.8

[101] Inomata T Eguchi Y Nakamura T Origin of Müllerian duct and its later development in relation to Wolffian duct and anogenital distance in the rat. Nihon Juigaku Zasshi. 1989;51:693–701. 2585924 10.1292/jvms1939.51.693

[102] Inomata T Eguchi Y Nakamura T Origin of Müllerian duct and its later developmental changes in relation to wolffian duct in bovine fetuses. Zentralbl Veterinarmed A. 1989;36:166–174. 2499995 10.1111/j.1439-0442.1989.tb00717.x

[103] Inomata T Inoue S Sugawara H Developmental changes in paramesonephric and mesonephric ducts and the external genitalia in swine fetuses during sexual differentiation. J Vet Med Sci. 1993;55:371–378. 8357908 10.1292/jvms.55.371

[104] Inomata T Ninomiya H Sakita K Developmental changes of Müllerian and Wolffian ducts in domestic cat fetuses. Exp Anim. 2009;58:41–45. 19151510 10.1538/expanim.58.41

[105] Paranko J Foidart JM Pelliniemi LJ Basement membrane in differentiating mesonephric and paramesonephric ducts of male and female rat fetuses. Differentiation. 1985;29:39–49. 4018459 10.1111/j.1432-0436.1985.tb00290.x

[106] Vazquez MD Bouchet P Foliguet B Gérard H Mallet JL Leheup B Differentiated aspect of female and male mouse mesonephroi. Int J Dev Biol. 1998;42:621–624. 9694634

[107] Vazquez MD Bouchet P Mallet JL Foliguet B Gérard H LeHeup B 3D reconstruction of the mouse's mesonephros. Anat Histol Embryol. 1998;27:283–287. 9818444 10.1111/j.1439-0264.1998.tb00194.x

[108] Mullen RD Behringer RR Molecular genetics of Müllerian duct formation, regression and differentiation. Sex Dev. 2014;8:281–296. 25033758 10.1159/000364935 PMC4378544

[109] Shaw G Renfree MB Wolffian duct development. Sex Dev. 2014;8:273–280. 24942390 10.1159/000363432

[110] Byskov AG Lintern-Moore S Follicle formation in the immature mouse ovary: the role of the rete ovarii. J Anat. 1973;116:207–217. 4783416 PMC1271596

[111] Löffler S Horn LC Weber W Spanel-Borowski K The transient disappearance of cytokeratin in human fetal and adult ovaries. Anat Embryol. 2000;201:207–215. 10664181 10.1007/s004290050019

[112] Byskov AG Does the rete ovarii act as a trigger for the onset of meiosis? Nature. 1974;252:396–397. 4431461 10.1038/252396a0

[113] Byskov AG Skakkebaek NE Stafanger G Peters H Influence of ovarian surface epithelium and rete ovarii on follicle formation. J Anat. 1977;123:77–86. 838624 PMC1234254

[114] Sawyer HR Smith P Heath DA Juengel JL Wakefield SJ McNatty KP Formation of ovarian follicles during fetal development in sheep. Biol Reprod. 2002;66:1134–1150. 11906935 10.1095/biolreprod66.4.1134

[115] Konishi I Fujii S Okamura H Parmley T Mori T Development of interstitial cells and ovigerous cords in the human fetal ovary: an ultrastructural study. J Anat. 1986;148:121–135. 3693081 PMC1261596

[116] Anderson RA Fulton N Cowan G Coutts S Saunders PT Conserved and divergent patterns of expression of DAZL, VASA and OCT4 in the germ cells of the human fetal ovary and testis. BMC Dev Biol. 2007;7:136. 18088417 10.1186/1471-213X-7-136 PMC2211489

[117] Kerr CL Hill CM Blumenthal PD Gearhart JD Expression of pluripotent stem cell markers in the human fetal ovary. Hum Reprod. 2008;23:589–599. 18203707 10.1093/humrep/dem411

[118] Burkhart MN Juengel JL Smith PR Morphological development and characterization of aromatase and estrogen receptors α and β in fetal ovaries of cattle from days 110 to 250. Anim Reprod Sci. 2010;117:43–54. 19299095 10.1016/j.anireprosci.2009.02.010

[119] van den Hurk R Dijkstra G van Mil FN Hulshof SC van den Ingh TS Distribution of the intermediate filament proteins vimentin, keratin, and desmin in the bovine ovary. Mol Reprod Dev. 1995;41:459–467. 7576613 10.1002/mrd.1080410408

[120] McNatty KP Fidler AE Juengel JL Growth and paracrine factors regulating follicular formation and cellular function. Mol Cell Endocrinol. 2000;163:11–20. 10963868 10.1016/s0303-7207(99)00235-x

[121] McNatty KP Smith P Hudson NL Development of the sheep ovary during fetal and early neonatal life and the effect of fecundity genes. J Reprod Fertil Suppl. 1995;49:123–135. 7623307

[122] Bullejos M Koopman P Germ cells enter meiosis in a rostro-caudal wave during development of the mouse ovary. Mol Reprod Dev. 2004;68:422–428. 15236325 10.1002/mrd.20105

[123] Rajah R Glaser EM Hirshfield AN The changing architecture of the neonatal rat ovary during histogenesis. Dev Dyn. 1992;194:177–192. 1467554 10.1002/aja.1001940303

[124] Hirshfield AN Heterogeneity of cell populations that contribute to the formation of primordial follicles in rats. Biol Reprod. 1992;47:466–472. 1511099 10.1095/biolreprod47.3.466

[125] Hirshfield AN DeSanti AM Patterns of ovarian cell proliferation in rats during the embryonic period and the first three weeks postpartum. Biol Reprod. 1995;53:1208–1221. 8527527 10.1095/biolreprod53.5.1208

[126] Mork L Maatouk DM McMahon JA Temporal differences in granulosa cell specification in the ovary reflect distinct follicle fates in mice. Biol Reprod. 2012;86:37. 21976597 10.1095/biolreprod.111.095208 PMC3290667

[127] Zheng W Zhang H Gorre N Risal S Shen Y Liu K Two classes of ovarian primordial follicles exhibit distinct developmental dynamics and physiological functions. Hum Mol Genet. 2014;23:920–928. 24087793 10.1093/hmg/ddt486 PMC3900105

[128] Rastetter RH Bernard P Palmer JS Marker genes identify three somatic cell types in the fetal mouse ovary. Dev Biol. 2014;394:242–252. 25158167 10.1016/j.ydbio.2014.08.013

[129] Pisarska MD Barlow G Kuo FT Minireview: roles of the forkhead transcription factor FOXL2 in granulosa cell biology and pathology. Endocrinology. 2011;152:1199–1208. 21248146 10.1210/en.2010-1041 PMC3206711

[130] Murdoch WJ Ovarian surface epithelium during ovulatory and anovulatory ovine estrous cycles. Anat Rec. 1994;240:322–326. 7825729 10.1002/ar.1092400305

[131] Auersperg N Wong AS Choi KC Kang SK Leung PC Ovarian surface epithelium: biology, endocrinology, and pathology. Endocr Rev. 2001;22:255–288. 11294827 10.1210/edrv.22.2.0422

[132] Wong AS Leung PC Role of endocrine and growth factors on the ovarian surface epithelium. J Obstet Gynaecol Res. 2007;33:3–16. 17212660 10.1111/j.1447-0756.2007.00478.x

[133] Murdoch WJ McDonnel AC Roles of the ovarian surface epithelium in ovulation and carcinogenesis. Reproduction. 2002;123:743–750. 12052228 10.1530/rep.0.1230743

[134] Byskov AG Differentiation of mammalian embryonic gonad. Physiol Rev. 1986;66:71–117. 3511481 10.1152/physrev.1986.66.1.71

[135] Kenngott RA Vermehren M Ebach K Sinowatz F The role of ovarian surface epithelium in folliculogenesis during fetal development of the bovine ovary: a histological and immunohistochemical study. Sex Dev. 2013;7:180–195. 23571709 10.1159/000348881

[136] Flesken-Nikitin A Hwang CI Cheng CY Michurina TV Enikolopov G Nikitin AY Ovarian surface epithelium at the junction area contains a cancer-prone stem cell niche. Nature. 2013;495:241–245. 23467088 10.1038/nature11979 PMC3982379

[137] Ng A Tan S Singh G Lgr5 marks stem/progenitor cells in ovary and tubal epithelia. Nat Cell Biol. 2014;16:745–757. 24997521 10.1038/ncb3000

[138] Szotek PP Chang HL Brennand K Normal ovarian surface epithelial label-retaining cells exhibit stem/progenitor cell characteristics. Proc Natl Acad Sci USA. 2008;105:12469–12473. 18711140 10.1073/pnas.0805012105 PMC2527935

[139] Usongo M Farookhi R β-Catenin/Tcf-signaling appears to establish the murine ovarian surface epithelium (OSE) and remains active in selected postnatal OSE cells. BMC Dev Biol. 2012;12:17. 22682531 10.1186/1471-213X-12-17 PMC3465187

[140] Bernard P Fleming A Lacombe A Harley VR Vilain E Wnt4 inhibits β-catenin/TCF signalling by redirecting β-catenin to the cell membrane. Biol Cell. 2008;100:167–177. 17976036 10.1042/BC20070072 PMC2670395

[141] Usongo M Li X Farookhi R Activation of the canonical WNT signaling pathway promotes ovarian surface epithelial proliferation without inducing β-catenin/Tcf-mediated reporter expression. Dev Dyn. 2013;242:291–300. 23239518 10.1002/dvdy.23919

[142] Gamwell LF Collins O Vanderhyden BC The mouse ovarian surface epithelium contains a population of LY6A (SCA-1) expressing progenitor cells that are regulated by ovulation-associated factors. Biol Reprod. 2012;87:80. 22914315 10.1095/biolreprod.112.100347

[143] Auersperg N The stem-cell profile of ovarian surface epithelium is reproduced in the oviductal fimbriae, with increased stem-cell marker density in distal parts of the fimbriae. Int J Gynecol Pathol. 2013;32:444–453. 23896717 10.1097/PGP.0b013e3182800ad5

[144] Young JM McNeilly AS Theca: the forgotten cell of the ovarian follicle. Reproduction. 2010;140:489–504. 20628033 10.1530/REP-10-0094

[145] Honda A Hirose M Hara K Isolation, characterization, and in vitro and in vivo differentiation of putative thecal stem cells. Proc Natl Acad Sci USA. 2007;104:12389–12394. 17636128 10.1073/pnas.0703787104 PMC1941479

[146] Rodgers HF Lavranos TC Vella CA Rodgers RJ Basal lamina and other extracellular matrix produced by bovine granulosa cells in anchorage-independent culture. Cell Tissue Res. 1995;282:463–471. 8581940 10.1007/BF00318878

[147] Irving-Rodgers HF Harland ML Rodgers RJ A novel basal lamina matrix of the stratified epithelium of the ovarian follicle. Matrix Biol. 2004;23:207–217. 15296935 10.1016/j.matbio.2004.05.008

[148] Hatzirodos N Nigro J Irving-Rodgers HF Glycomic analyses of ovarian follicles during development and atresia. Matrix Biol. 2012;31:45–56. 22057033 10.1016/j.matbio.2011.10.002 PMC3657699

[149] Lee YM Kumar BM Lee JH Characterisation and differentiation of porcine ovarian theca-derived multipotent stem cells. Vet J. 2013;197:761–768. 23702282 10.1016/j.tvjl.2013.04.011

[150] Brannstrom M Mayrhofer G Robertson SA Localization of leukocyte subsets in the rat ovary during periovulatory period. Biol Reprod. 1993;48:277–286. 8439617 10.1095/biolreprod48.2.277

[151] Rodgers RJ Lavranos TC van Wezel IL Irving-Rodgers HF Development of the ovarian follicular epithelium. Mol Cell Endocrinol. 1999;151:171–179. 10411332 10.1016/s0303-7207(99)00087-8

[152] Lavranos TC Rodgers HF Bertoncello I Rodgers RJ Anchorage-independent culture of bovine granulosa cells: the effects of basic fibroblast growth factor and dibutyryl cAMP on cell division and differentiation. Exp Cell Res. 1994;211:245–251. 8143770 10.1006/excr.1994.1084

[153] Lavranos TC Rodgers RJ An assay of tritiated thymidine incorporation into DNA by cells cultured under anchorage-independent conditions. Anal Biochem. 1994;223:325–327. 7887480 10.1006/abio.1994.1593

[154] Lavranos TC O'Leary PC Rodgers RJ Effects of insulin-like growth factors and binding protein 1 on bovine granulosa cell division in anchorage-independent culture. J Reprod Fertil. 1996;107:221–228. 8882288 10.1530/jrf.0.1070221

[155] Rodgers RJ Vella CA Rodgers HF Scott K Lavranos TC Production of extracellular matrix, fibronectin and steroidogenic enzymes, and growth of bovine granulosa cells in anchorage-independent culture. Reprod Fertil Dev. 1996;8:249–257. 8726863 10.1071/rd9960249

[156] Lavranos TC Mathis JM Latham SE Kalionis B Shay JW Rodgers RJ Evidence for ovarian granulosa stem cells: telomerase activity and localization of the telomerase ribonucleic acid component in bovine ovarian follicles. Biol Reprod. 1999;61:358–366. 10411512 10.1095/biolreprod61.2.358

[157] Wells DN Misica PM Tervit HR Production of cloned calves following nuclear transfer with cultured adult mural granulosa cells. Biol Reprod. 1999;60:996–1005. 10084977 10.1095/biolreprod60.4.996

[158] Shin T Kraemer D Pryor J A cat cloned by nuclear transplantation. Nature. 2002;415:859. 11859353 10.1038/nature723

[159] Kossowska-Tomaszczuk K De Geyter C De Geyter M The multipotency of luteinizing granulosa cells collected from mature ovarian follicles. Stem Cells. 2009;27:210–219. 19224509 10.1634/stemcells.2008-0233

[160] Merkwitz C Ricken AM Lösche A Sakurai M Spanel-Borowski K Progenitor cells harvested from bovine follicles become endothelial cells. Differentiation. 2010;79:203–210. 20303645 10.1016/j.diff.2010.02.004

[161] Dzafic E Stimpfel M Novakovic S Cerkovnik P Virant-Klun I Expression of mesenchymal stem cells-related genes and plasticity of aspirated follicular cells obtained from infertile women. Biomed Res Int. 2014;2014:508216. 24724084 10.1155/2014/508216 PMC3958784

[162] Antczak M Van Blerkom J The vascular character of ovarian follicular granulosa cells: phenotypic and functional evidence for an endothelial-like cell population. Hum Reprod. 2000;15:2306–2318. 11056124 10.1093/humrep/15.11.2306

[163] Kossowska-Tomaszczuk K De Geyter C Cells with stem cell characteristics in somatic compartments of the ovary. Biomed Res Int. 2013;2013:310859. 23484108 10.1155/2013/310859 PMC3591217

[164] Lan CW Chen MJ Jan PS Chen HF Ho HN Differentiation of human embryonic stem cells into functional ovarian granulosa-like cells. J Clin Endocrinol Metab. 2013;98:3713–3723. 23884780 10.1210/jc.2012-4302

[165] Kumar TR Wang Y Lu N Matzuk MM Follicle stimulating hormone is required for ovarian follicle maturation but not male fertility. Nat Genet. 1997;15:201–204. 9020850 10.1038/ng0297-201

[166] Dong J Albertini DF Nishimori K Kumar TR Lu N Matzuk MM Growth differentiation factor-9 is required during early ovarian folliculogenesis. Nature. 1996;383:531–535. 8849725 10.1038/383531a0

[167] Dube JL Wang P Elvin J Lyons KM Celeste AJ Matzuk MM The bone morphogenetic protein 15 gene is X-linked and expressed in oocytes. Mol Endocrinol. 1998;12:1809–1817. 9849956 10.1210/mend.12.12.0206

[168] Sugiura K Su YQ Diaz FJ Oocyte-derived BMP15 and FGFs cooperate to promote glycolysis in cumulus cells. Development. 2007;134:2593–2603. 17553902 10.1242/dev.006882

[169] Oron G Fisch B Zhang XY Fibroblast growth factor 10 in human ovaries. Reprod Biomed Online. 2012;25:396–401. 22877940 10.1016/j.rbmo.2012.07.002

[170] Buratini J Jr Pinto MG Castilho AC Expression and function of fibroblast growth factor 10 and its receptor, fibroblast growth factor receptor 2B, in bovine follicles. Biol Reprod. 2007;77:743–750. 17582010 10.1095/biolreprod.107.062273

[171] Zhang M Su YQ Sugiura K Wigglesworth K Xia G Eppig JJ Estradiol promotes and maintains cumulus cell expression of natriuretic peptide receptor 2 (NPR2) and meiotic arrest in mouse oocytes in vitro. Endocrinology. 2011;152:4377–4385. 21914782 10.1210/en.2011-1118 PMC3199003

[172] Ochsner SA Russell DL Day AJ Breyer RM Richards JS Decreased expression of tumor necrosis factor-α-stimulated gene 6 in cumulus cells of the cyclooxygenase-2 and EP2 null mice. Endocrinology. 2003;144:1008–1019. 12586778 10.1210/en.2002-220435

[173] Rider CC Mulloy B Bone morphogenetic protein and growth differentiation factor cytokine families and their protein antagonists. Biochem J. 2010;429:1–12. 20545624 10.1042/BJ20100305

[174] Watson LN Mottershead DG Dunning KR Robker RL Gilchrist RB Russell DL Heparan sulfate proteoglycans regulate responses to oocyte paracrine signals in ovarian follicle morphogenesis. Endocrinology. 2012;153:4544–4555. 22759380 10.1210/en.2012-1181

[175] Juengel JL McNatty KP The role of proteins of the transforming growth factor-β superfamily in the intraovarian regulation of follicular development. Hum Reprod Update. 2005;11:143–160. 15705960 10.1093/humupd/dmh061

[176] Mao GK Li JX Bian FH Gap junction -mediated cAMP movement between oocytes and somatic cells. Front Biosci (Elite Ed). 2013;5:755–767. 23277030 10.2741/e656

[177] Macaulay AD Gilbert I Caballero J The gametic synapse: RNA transfer to the bovine oocyte. Biol Reprod. 2014;91:90. 25143353 10.1095/biolreprod.114.119867

[178] Pirino G Wescott MP Donovan PJ Protein kinase A regulates resumption of meiosis by phosphorylation of Cdc25B in mammalian oocytes. Cell Cycle. 2009;8:665–670. 19223768 10.4161/cc.8.4.7846

[179] Richard S Baltz JM Prophase I arrest of mouse oocytes mediated by natriuretic peptide precursor C requires GJA1 (connexin-43) and GJA4 (connexin-37) gap junctions in the antral follicle and cumulus-oocyte complex. Biol Reprod. 2014;90:137. 24804968 10.1095/biolreprod.114.118505

[180] Wigglesworth K Lee KB O'Brien MJ Peng J Matzuk MM Eppig JJ Bidirectional communication between oocytes and ovarian follicular somatic cells is required for meiotic arrest of mammalian oocytes. Proc Natl Acad Sci USA. 2013;110:E3723–E3729. 23980176 10.1073/pnas.1314829110 PMC3785791

[181] Sugiura K Pendola FL Eppig JJ Oocyte control of metabolic cooperativity between oocytes and companion granulosa cells: energy metabolism. Dev Biol. 2005;279:20–30. 15708555 10.1016/j.ydbio.2004.11.027

[182] Chen J Torcia S Xie F Somatic cells regulate maternal mRNA translation and developmental competence of mouse oocytes. Nat Cell Biol. 2013;15:1415–1423. 24270888 10.1038/ncb2873 PMC4066669

[183] Russell DL Robker RL Molecular mechanisms of ovulation: co-ordination through the cumulus complex. Hum Reprod Update. 2007;13:289–312. 17242016 10.1093/humupd/dml062

[184] Tsafriri A Ovulation as a tissue remodelling process. Proteolysis and cumulus expansion. Adv Exp Med Biol. 1995;377:121–140. 7484419 10.1007/978-1-4899-0952-7_8

[185] Richards JS Russell DL Ochsner S Espey LL Ovulation: new dimensions and new regulators of the inflammatory-like response. Annu Rev Physiol. 2002;64:69–92. 11826264 10.1146/annurev.physiol.64.081501.131029

[186] Hsieh M Lee D Panigone S Luteinizing hormone-dependent activation of the epidermal growth factor network is essential for ovulation. Mol Cell Biol. 2007;27:1914–1924. 17194751 10.1128/MCB.01919-06 PMC1820474

[187] Park JY Su YQ Ariga M Law E Jin SL Conti M EGF-like growth factors as mediators of LH action in the ovulatory follicle. Science. 2004;303:682–684. 14726596 10.1126/science.1092463

[188] Fan HY Liu Z Shimada M MAPK3/1 (ERK1/2) in ovarian granulosa cells are essential for female fertility. Science. 2009;324:938–941. 19443782 10.1126/science.1171396 PMC2847890

[189] Duggavathi R Volle DH Mataki C Liver receptor homolog 1 is essential for ovulation. Genes Dev. 2008;22:1871–1876. 18628394 10.1101/gad.472008 PMC2492734

[190] Lim H Paria BC Das SK Multiple female reproductive failures in cyclooxygenase 2-deficient mice. Cell. 1997;91:197–208. 9346237 10.1016/s0092-8674(00)80402-x

[191] Peters MW Pursley JR Smith GW Inhibition of intrafollicular PGE2 synthesis and ovulation following ultrasound-mediated intrafollicular injection of the selective cyclooxygenase-2 inhibitor NS-398 in cattle. J Anim Sci. 2004;82:1656–1662. 15216991 10.2527/2004.8261656x

[192] Tsafriri A Reich R Molecular aspects of mammalian ovulation. Exp Clin Endocrinol Diabetes. 1999;107:1–11. 10077349 10.1055/s-0029-1212066

[193] Takahashi T Morrow JD Wang H Dey SK Cyclooxygenase-2-derived prostaglandin E(2) directs oocyte maturation by differentially influencing multiple signaling pathways. J Biol Chem. 2006;281:37117–37129. 17023426 10.1074/jbc.M608202200

[194] Duffy DM McGinnis LK Vandevoort CA Christenson LK Mammalian oocytes are targets for prostaglandin E2 (PGE2) action. Reprod Biol Endocrinol. 2010;8:131. 21040553 10.1186/1477-7827-8-131 PMC2988801

[195] Peluffo MC Stanley J Braeuer N A prostaglandin E2 receptor antagonist prevents pregnancies during a preclinical contraceptive trial with female macaques. Hum Reprod. 2014;29:1400–1412. 24781425 10.1093/humrep/deu083 PMC4059334

[196] Robker RL Akison LK Russell DL Control of oocyte release by progesterone receptor-regulated gene expression. Nucl Recept Signal. 2009;7:e012. 20087433 10.1621/nrs.07012 PMC2807638

[197] Conneely OM Mulac-Jericevic B Lydon JP De Mayo FJ Reproductive functions of the progesterone receptor isoforms: lessons from knock-out mice. Mol Cell Endocrinol. 2001;179:97–103. 11420134 10.1016/s0303-7207(01)00465-8

[198] Marions L Hultenby K Lindell I Sun X Ståbi B Gemzell Danielsson K Emergency contraception with mifepristone and levonorgestrel: mechanism of action. Obstet Gynecol. 2002;100:65–71. 12100805 10.1016/s0029-7844(02)02006-9

[199] Stratton P Hartog B Hajizadeh N A single mid-follicular dose of CDB-2914, a new antiprogestin, inhibits folliculogenesis and endometrial differentiation in normally cycling women. Hum Reprod. 2000;15:1092–1099. 10783359 10.1093/humrep/15.5.1092

[200] Bridges PJ Komar CM Fortune JE Gonadotropin-induced expression of messenger ribonucleic acid for cyclooxygenase-2 and production of prostaglandins E and F2α in bovine preovulatory follicles are regulated by the progesterone receptor. Endocrinology. 2006;147:4713–4722. 16825323 10.1210/en.2005-1575

[201] Gaytán F Bellido C Gaytán M Morales C Sánchez-Criado JE Differential effects of RU486 and indomethacin on follicle rupture during the ovulatory process in the rat. Biol Reprod. 2003;69:99–105. 12606368 10.1095/biolreprod.102.013755

[202] Irving-Rodgers HF Catanzariti KD Aspden WJ D'Occhio MJ Rodgers RJ Remodeling of extracellular matrix at ovulation of the bovine ovarian follicle. Mol Reprod Dev. 2006;73:1292–1302. 16865721 10.1002/mrd.20580

[203] Brown HM Dunning KR Robker RL ADAMTS1 cleavage of versican mediates essential structural remodeling of the ovarian follicle and cumulus-oocyte matrix during ovulation in mice. Biol Reprod. 2010;83:549–557. 20592310 10.1095/biolreprod.110.084434

[204] Brown HM Russell DL Blood and lymphatic vasculature in the ovary: development, function and disease. Hum Reprod Update. 2014;20:29–39. 24097804 10.1093/humupd/dmt049

[205] Ohnishi J Ohnishi E Shibuya H Takahashi T Functions for proteinases in the ovulatory process. Biochim Biophys Acta. 2005;1751:95–109. 15950557 10.1016/j.bbapap.2005.05.002

[206] Curry TE Jr ADAMTS1 and versican: partners in ovulation and fertilization. Biol Reprod. 2010;83:505–506. 20631403 10.1095/biolreprod.110.087056

[207] Peluffo MC Murphy MJ Baughman ST Stouffer RL Hennebold JD Systematic analysis of protease gene expression in the rhesus macaque ovulatory follicle: metalloproteinase involvement in follicle rupture. Endocrinology. 2011;152:3963–3974. 21791558 10.1210/en.2011-1172 PMC3176652

[208] Puttabyatappa M Jacot TA Al-Alem LF Ovarian membrane-type matrix metalloproteinases: induction of MMP14 and MMP16 during the periovulatory period in the rat, macaque, and human. Biol Reprod. 2014;91:34. 24920038 10.1095/biolreprod.113.115717 PMC4435413

[209] Chen L Russell PT Larsen WJ Functional significance of cumulus expansion in the mouse: roles for the preovulatory synthesis of hyaluronic acid within the cumulus mass. Mol Reprod Dev. 1993;34:87–93. 8418823 10.1002/mrd.1080340114

[210] Chen L Mao SJ Larsen WJ Identification of a factor in fetal bovine serum that stabilizes the cumulus extracellular matrix. A role for a member of the inter-α-trypsin inhibitor family. J Biol Chem. 1992;267:12380–12386. 1376324

[211] Chen L Mao SJ McLean LR Powers RW Larsen WJ Proteins of the inter-α-trypsin inhibitor family stabilize the cumulus extracellular matrix through their direct binding with hyaluronic acid. J Biol Chem. 1994;269:28282–28287. 7525572

[212] Hess KA Chen L Larsen WJ Inter-α-inhibitor binding to hyaluronan in the cumulus extracellular matrix is required for optimal ovulation and development of mouse oocytes. Biol Reprod. 1999;61:436–443. 10411524 10.1095/biolreprod61.2.436

[213] Carrette O Nemade RV Day AJ Brickner A Larsen WJ TSG-6 is concentrated in the extracellular matrix of mouse cumulus oocyte complexes through hyaluronan and inter-α-inhibitor binding. Biol Reprod. 2001;65:301–308. 11420253 10.1095/biolreprod65.1.301

[214] Russell DL Salustri A Extracellular matrix of the cumulus-oocyte complex. Semin Reprod Med. 2006;24:217–227. 16944419 10.1055/s-2006-948551

[215] Varani S Elvin JA Yan C Knockout of pentraxin 3, a downstream target of growth differentiation factor-9, causes female subfertility. Mol Endocrinol. 2002;16:1154–1167. 12040004 10.1210/mend.16.6.0859

[216] Salustri A Garlanda C Hirsch E PTX3 plays a key role in the organization of the cumulus oophorus extracellular matrix and in in vivo fertilization. Development. 2004;131:1577–1586. 14998931 10.1242/dev.01056

[217] Russell DL Ochsner SA Hsieh M Mulders S Richards JS Hormone-regulated expression and localization of versican in the rodent ovary. Endocrinology. 2003;144:1020–1031. 12586779 10.1210/en.2002-220434

[218] Rodgers RJ Irving-Rodgers HF Russell DL Extracellular matrix of the developing ovarian follicle. Reproduction. 2003;126:415–424. 14525524 10.1530/rep.0.1260415

[219] Russell DL Doyle KM Ochsner SA Sandy JD Richards JS Processing and localization of ADAMTS-1 and proteolytic cleavage of versican during cumulus matrix expansion and ovulation. J Biol Chem. 2003;278:42330–42339. 12907688 10.1074/jbc.M300519200

[220] Zhuo L Yoneda M Zhao M Defect in SHAP-hyaluronan complex causes severe female infertility. A study by inactivation of the bikunin gene in mice. J Biol Chem. 2001;276:7693–7696. 11145954 10.1074/jbc.C000899200

[221] ØDum L Andersen CY Jessen TE Characterization of the coupling activity for the binding of inter-α-trypsin inhibitor to hyaluronan in human and bovine follicular fluid. Reproduction. 2002;124:249–257. 12141938 10.1530/rep.0.1240249

[222] Scarchilli L Camaioni A Bottazzi B PTX3 interacts with inter-α-trypsin inhibitor: implications for hyaluronan organization and cumulus oophorus expansion. J Biol Chem. 2007;282:30161–30170. 17675295 10.1074/jbc.M703738200

[223] Colón E Shytuhina A Cowman MK Transfer of inter-α-inhibitor heavy chains to hyaluronan by surface-linked hyaluronan-TSG-6 complexes. J Biol Chem. 2009;284:2320–2331. 19033448 10.1074/jbc.M807183200

[224] Akison LK Alvino ER Dunning KR Robker RL Russell DL Transient invasive migration in mouse cumulus oocyte complexes induced at ovulation by luteinizing hormone. Biol Reprod. 2012;86:125. 22238281 10.1095/biolreprod.111.097345

[225] Espey LL Ovulation as an inflammatory reaction–a hypothesis. Biol Reprod. 1980;22:73–106. 6991013 10.1095/biolreprod22.1.73

[226] Papacleovoulou G Critchley HO Hillier SG Mason JI IL1α and IL4 signalling in human ovarian surface epithelial cells. J Endocrinol. 2011;211:273–283. 21903865 10.1530/JOE-11-0081

[227] Papacleovoulou G Hogg K Fegan KS Critchley HO Hillier SG Mason JI Regulation of 3β-hydroxysteroid dehydrogenase type 1 and type 2 gene expression and function in the human ovarian surface epithelium by cytokines. Mol Hum Reprod. 2009;15:379–392. 19414525 10.1093/molehr/gap022

[228] Takehara Y Dharmarajan AM Kaufman G Wallach EE Effect of interleukin-1 β on ovulation in the in vitro perfused rabbit ovary. Endocrinology. 1994;134:1788–1793. 8137743 10.1210/endo.134.4.8137743

[229] Liu Z de Matos DG Fan HY Shimada M Palmer S Richards JS Interleukin-6: an autocrine regulator of the mouse cumulus cell-oocyte complex expansion process. Endocrinology. 2009;150:3360–3368. 19299453 10.1210/en.2008-1532 PMC2703543

[230] Gérard N Caillaud M Martoriati A Goudet G Lalmanach AC The interleukin-1 system and female reproduction. J Endocrinol. 2004;180:203–212. 14765973 10.1677/joe.0.1800203

[231] Tam KK Russell DL Peet DJ Hormonally regulated follicle differentiation and luteinization in the mouse is associated with hypoxia inducible factor activity. Mol Cell Endocrinol. 2010;327:47–55. 20600586 10.1016/j.mce.2010.06.008

[232] Van der Hoek KH Maddocks S Woodhouse CM van Rooijen N Robertson SA Norman RJ Intrabursal injection of clodronate liposomes causes macrophage depletion and inhibits ovulation in the mouse ovary. Biol Reprod. 2000;62:1059–1066. 10727278 10.1095/biolreprod62.4.1059

[233] Araki M Fukumatsu Y Katabuchi H Shultz LD Takahashi K Okamura H Follicular development and ovulation in macrophage colony-stimulating factor-deficient mice homozygous for the osteopetrosis (op) mutation. Biol Reprod. 1996;54:478–484. 8788202 10.1095/biolreprod54.2.478

[234] Cohen-Fredarow A Tadmor A Raz T Ovarian dendritic cells act as a double-edged pro-ovulatory and anti-inflammatory sword. Mol Endocrinol. 2014;28:1039–1054. 24825398 10.1210/me.2013-1400 PMC5414831

[235] Stocco C Telleria C Gibori G The molecular control of corpus luteum formation, function, and regression. Endocr Rev. 2007;28:117–149. 17077191 10.1210/er.2006-0022

[236] Madan P Bridges PJ Komar CM Expression of messenger RNA for ADAMTS subtypes changes in the periovulatory follicle after the gonadotropin surge and during luteal development and regression in cattle. Biol Reprod. 2003;69:1506–1514. 12855604 10.1095/biolreprod.102.013714

[237] Murdoch WJ Gottsch ML Proteolytic mechanisms in the ovulatory folliculo-luteal transformation. Connect Tissue Res. 2003;44:50–57. 12945804

[238] Irving-Rodgers HF Friden BE Morris SE Extracellular matrix of the human cyclic corpus luteum. Mol Hum Reprod. 2006;12:525–534. 16870952 10.1093/molehr/gal060

[239] Silvester LM Luck MR Distribution of extracellular matrix components in the developing ruminant corpus luteum: a wound repair hypothesis for luteinization. J Reprod Fertil. 1999;116:187–198. 10505069 10.1530/jrf.0.1160187

[240] Irving-Rodgers HF Hummitzsch K Murdiyarso LS Dynamics of extracellular matrix in ovarian follicles and corpora lutea of mice. Cell Tissue Res. 2010;339:613–624. 20033213 10.1007/s00441-009-0905-8 PMC2831189

[241] Matsushima T Fukuda Y Tsukada K Yamanaka N The extracellular matrices and vascularization of the developing corpus luteum in rats. J Submicrosc Cytol Pathol. 1996;28:441–455. 8933731

[242] Irving-Rodgers HF Roger J Luck MR Rodgers RJ Extracellular matrix of the corpus luteum. Semin Reprod Med. 2006;24:242–250. 16944421 10.1055/s-2006-948553

[243] Lee JM Dedhar S Kalluri R Thompson EW The epithelial-mesenchymal transition: new insights in signaling, development, and disease. J Cell Biol. 2006;172:973–981. 16567498 10.1083/jcb.200601018 PMC2063755

[244] Rodgers RJ Irving-Rodgers HF van Wezel IL Krupa M Lavranos TC Dynamics of the membrana granulosa during expansion of the ovarian follicular antrum. Mol Cell Endocrinol. 2001;171:41–48. 11165009 10.1016/s0303-7207(00)00430-5

[245] Rodgers RJ Irving Rodgers HF Extracellular matrix of the bovine ovarian membrana granulosa. Mol Cell Endocrinol. 2002;191:57–64. 12044919 10.1016/s0303-7207(02)00057-6

[246] Luck MR Rodgers RJ Findlay JK Secretion and gene expression of inhibin, oxytocin and steroid hormones during the in vitro differentiation of bovine granulosa cells. Reprod Fertil Dev. 1990;2:11–25. 1692150 10.1071/rd9900011

[247] Luck MR Greatly elevated and sustained secretion of oxytocin by bovine granulosa cells in serum-free culture. J Exp Zool. 1989;251:361–366. 2769209 10.1002/jez.1402510313

[248] Alexopoulos E Shahid J Ongley HZ Richardson MC Luteinized human granulosa cells are associated with endogenous basement membrane-like components in culture. Mol Hum Reprod. 2000;6:324–330. 10729314 10.1093/molehr/6.4.324

[249] Richardson MC Slack C Stewart IJ Rearrangement of extracellular matrix during cluster formation by human luteinising granulosa cells in culture. J Anat. 2000;196:243–248. 10739020 10.1046/j.1469-7580.2000.19620243.x PMC1468057

[250] Zhao Y Luck MR Collagen and its remodelling in ruminants follicles/corpora lutea. Adv Contracept Deliv Syst. 1996;12:41–49.

[251] Adams EC Hertig AT Studies on the human corpus luteum. II. Observations on the ultrastructure of luteal cells during pregnancy. J Cell Biol. 1969;41:716–735. 5768871 10.1083/jcb.41.3.716 PMC2107828

[252] O'Shea JD Rodgers RJ D'Occhio MJ Cellular composition of the cyclic corpus luteum of the cow. J Reprod Fertil. 1989;85:483–487. 2703988 10.1530/jrf.0.0850483

[253] Wang LJ Pascoe V Petrucco OM Norman RJ Distribution of leukocyte subpopulations in the human corpus luteum. Hum Reprod. 1992;7:197–202. 1533647 10.1093/oxfordjournals.humrep.a137616

[254] Brännström M Giesecke L Moore IC van den Heuvel CJ Robertson SA Leukocyte subpopulations in the rat corpus luteum during pregnancy and pseudopregnancy. Biol Reprod. 1994;50:1161–1167. 8025173 10.1095/biolreprod50.5.1161

[255] Kirsch TM Friedman AC Vogel RL Flickinger GL Macrophages in corpora lutea of mice: characterization and effects on steroid secretion. Biol Reprod. 1981;25:629–638. 7030418 10.1095/biolreprod25.3.629

[256] Pate JL Landis Keyes P Immune cells in the corpus luteum: friends or foes? Reproduction. 2001;122:665–676. 11690526 10.1530/rep.0.1220665

[257] Halme J Hammond MG Syrop CH Talbert LM Peritoneal macrophages modulate human granulosa-luteal cell progesterone production. J Clin Endocrinol Metab. 1985;61:912–916. 4044779 10.1210/jcem-61-5-912

[258] Castro A Castro O Troncoso JL Luteal leukocytes are modulators of the steroidogenic process of human mid-luteal cells. Hum Reprod. 1998;13:1584–1589. 9688396 10.1093/humrep/13.6.1584

[259] Care AS Diener KR Jasper MJ Brown HM Ingman WV Robertson SA Macrophages regulate corpus luteum development during embryo implantation in mice. J Clin Invest. 2013;123:3472–3487. 23867505 10.1172/JCI60561 PMC3726148

[260] Poole DH Pate JL Luteal microenvironment directs resident T lymphocyte function in cows. Biol Reprod. 2012;86:29. 21976598 10.1095/biolreprod.111.092296

[261] Stouffer RL Bishop CV Bogan RL Xu F Hennebold JD Endocrine and local control of the primate corpus luteum. Reprod Biol. 2013;13:259–271. 24287034 10.1016/j.repbio.2013.08.002 PMC4001828

[262] Pate JL Johnson-Larson CJ Ottobre JS Life or death decisions in the corpus luteum. Reprod Domest Anim. 2012;47(suppl 4):297–303. 22827384 10.1111/j.1439-0531.2012.02089.x

[263] McCracken JA Custer EE Lamsa JC Luteolysis: a neuroendocrine-mediated event. Physiol Rev. 1999;79:263–323. 10221982 10.1152/physrev.1999.79.2.263

[264] Vu HV Lee S Acosta TJ Yoshioka S Abe H Okuda K Roles of prostaglandin F2α and hydrogen peroxide in the regulation of copper/zinc superoxide dismutase in bovine corpus luteum and luteal endothelial cells. Reprod Biol Endocrinol. 2012;10:87. 23101731 10.1186/1477-7827-10-87 PMC3545964

[265] Diaz FJ Wiltbank MC Acquisition of luteolytic capacity involves differential regulation by prostaglandin F2α of genes involved in progesterone biosynthesis in the porcine corpus luteum. Domest Anim Endocrinol. 2005;28:172–189. 15713365 10.1016/j.domaniend.2004.08.002

[266] Zhang X Li J Liu J Luo H Gou K Cui S Prostaglandin F2α upregulates Slit/Robo expression in mouse corpus luteum during luteolysis. J Endocrinol. 2013;218:299–310. 23814012 10.1530/JOE-13-0088

[267] Dickinson RE Myers M Duncan WC Novel regulated expression of the SLIT/ROBO pathway in the ovary: possible role during luteolysis in women. Endocrinology. 2008;149:5024–5034. 18566128 10.1210/en.2008-0204

[268] Robertson SA Regulatory T cells in the corpus luteum–new players in fertility control? Biol Reprod. 2012;86:26. 22174024 10.1095/biolreprod.111.098301

[269] Devoto L Kohen P Vega M Control of human luteal steroidogenesis. Mol Cell Endocrinol. 2002;186:137–141. 11900886 10.1016/s0303-7207(01)00654-2

[270] Erlebacher A Zhang D Parlow AF Glimcher LH Ovarian insufficiency and early pregnancy loss induced by activation of the innate immune system. J Clin Invest. 2004;114:39–48. 15232610 10.1172/JCI20645 PMC437968

[271] Da Silva-Buttkus P Jayasooriya GS Mora JM Effect of cell shape and packing density on granulosa cell proliferation and formation of multiple layers during early follicle development in the ovary. J Cell Sci. 2008;121:3890–3900. 19001500 10.1242/jcs.036400

[272] Rodgers RJ Irving-Rodgers HF Morphological classification of bovine ovarian follicles. Reproduction. 2010;139:309–318. 19786400 10.1530/REP-09-0177

